# Distributed Wi-Fi-Based System for Monitoring the Condition of Building Structures

**DOI:** 10.3390/s26134217

**Published:** 2026-07-03

**Authors:** Nurbol Kaliaskarov, Ulan Yessenzholov, Ruslan Mekhtiyev, Elena Neshina, Marianella Gavrilova, Gulzat Mashrapova, Zhaina Zhaxylyk

**Affiliations:** 1 Department of Radio Electronics and Telecommunication Technologies, Faculty of Energy, Automation and Telecommunications, Abylkas Saginov Karaganda Technical University, Karaganda 100027, Kazakhstan; n.kaliaskarov@ktu.edu.kz (N.K.); m.gavrilova@ktu.edu.kz (M.G.); g.mashrapova@ktu.edu.kz (G.M.); zh.zhaksylyk@ktu.edu.kz (Z.Z.); 2 Department of Radio Engineering, Electronics and Telecommunications, Institute of Physical and Technical Sciences, L.N. Gumilyov Eurasian National University, Astana 010008, Kazakhstan; 3 Department of Industrial Process Automation, Faculty of Energy, Automation and Telecommunications, Abylkas Saginov Karaganda Technical University, Karaganda 100027, Kazakhstan; ruslanmekhtiyev@gmail.com; 4 Department of Energy Systems, Faculty of Energy, Automation and Telecommunications, Abylkas Saginov Karaganda Technical University, Karaganda 100027, Kazakhstan; e.neshina@ktu.edu.kz

**Keywords:** structural health monitoring (SHM), wireless sensor networks (WSNs), IEEE 802.11, distributed monitoring, sustainable development, remote sensing, structural diagnostics

## Abstract

This paper presents the development and experimental validation of a Wi-Fi-based distributed system for structural health monitoring of building structures. The proposed system employs a hybrid mesh/ad hoc architecture, enabling autonomous sensor nodes to communicate via IEEE 802.11 without requiring a centralized wired infrastructure. A distributed monitoring architecture, a data transmission algorithm, and a multi-sensor platform integrating distance, magnetometric, and environmental sensors were developed. A mathematical network model was established to analyze the communication characteristics and ensure reliable data exchange in the distributed system. The proposed approach was validated through 500 consecutive experimental measurements and a comparative analysis of wired and wireless data acquisition. The results demonstrated reliable wireless communication without data loss while preserving the temporal characteristics of the measured signals. The distance sensors and the DHT22 temperature sensor achieved the highest agreement with the wired reference based on the MAE and RMSE metrics, whereas the magnetometric sensors and humidity measurements exhibited moderate variability associated with sensor sensitivity and transmission conditions. The obtained results confirm that the proposed Wi-Fi-based distributed architecture provides a stable, scalable, and fault-tolerant solution for structural health monitoring. The developed system can also be adapted for environmental monitoring, energy systems, and other smart infrastructure applications.

## 1. Introduction

Structural health monitoring (SHM) has become one of the key technologies for ensuring the safe operation of engineering structures throughout their service life. Continuous monitoring of structural parameters allows the condition of buildings, bridges, and other critical infrastructure to be assessed without interrupting their operation. Early detection of structural degradation, excessive deformation, or abnormal environmental influences makes it possible to reduce maintenance costs, prevent emergency situations, and improve the overall reliability of engineering facilities. As infrastructure continues to age and construction projects become increasingly complex, the demand for intelligent monitoring systems capable of providing reliable real-time information has grown significantly.

Conventional SHM systems are predominantly based on wired sensor networks. Such systems provide stable communication and high measurement reliability; however, their practical implementation is often associated with considerable installation costs, complicated maintenance procedures, and limited flexibility when additional sensing points must be installed. Cable routing becomes particularly difficult in large buildings, bridges, industrial facilities, and structures with restricted access. These limitations have stimulated the rapid development of wireless sensor networks (WSNs), which simplify installation, reduce infrastructure requirements, and enable distributed monitoring of structural elements located over large areas.

During the past two decades, WSNs have evolved from experimental laboratory platforms into practical monitoring systems capable of supporting large numbers of sensing nodes. Comprehensive studies by Worden, Farrar, Manson, Park, Fan, Qiao, Balageas, Fritzen, Güemes, and other researchers established the theoretical foundations of structural health monitoring and demonstrated the potential of distributed sensing technologies for engineering diagnostics [1,2,3,4,5]. Subsequent review papers summarized the advantages of wireless monitoring systems, including simplified deployment, local data processing, and improved scalability, while also identifying several unresolved problems related to synchronization, energy consumption, communication reliability, and long-term operation [6,7]. More recent surveys further emphasized that many existing SHM platforms remain limited to laboratory-scale demonstrations, whereas issues such as reliable transmission of high-frequency measurement data, efficient network management, and practical deployment in large engineering structures still require further investigation [8,9].

Among existing wireless communication technologies, Wi-Fi has attracted increasing attention because it combines high transmission capacity with almost universal availability. Unlike many specialized low-power protocols, Wi-Fi is already integrated into modern communication infrastructure and supports direct interaction with cloud services, remote databases, and edge-computing platforms. At the same time, Wi-Fi has also evolved beyond its traditional communication role. Recent studies have demonstrated that channel state information (CSI) and received signal strength indicator (RSSI) measurements can be used for sensing applications, including motion detection, activity recognition, and environmental monitoring. The possibility of processing CSI data on inexpensive embedded devices, such as the ESP32 platform, has further expanded the range of potential monitoring applications [10].

Despite these promising developments, several important challenges remain unresolved. The quality of CSI measurements strongly depends on environmental conditions, including temperature variations, ventilation, surrounding objects, and human movement. In addition, access to CSI information depends on hardware platforms and firmware implementations, while reliable interpretation of long-term measurements often requires repeated calibration and sophisticated signal-processing algorithms. These factors significantly complicate the use of Wi-Fi sensing as a robust tool for structural diagnostics under practical operating conditions [11].

Practical implementation of SHM systems in buildings, bridges, and other civil structures has also revealed limitations that extend beyond sensing technologies themselves. Field deployments have shown that conventional centralized monitoring architectures often suffer from high installation costs, communication delays, limited scalability, and reduced flexibility during maintenance or network expansion. The dependence on fixed communication infrastructure additionally increases system vulnerability to local failures, particularly in large distributed monitoring networks [12]. These limitations motivate the development of more flexible wireless architectures capable of combining reliable communication with simple deployment and autonomous operation.

Recent studies have proposed a wide variety of approaches for improving structural health monitoring through the integration of advanced sensing technologies, distributed communication systems, and intelligent data-processing methods. Multi-sensor monitoring platforms have become one of the dominant research directions because they enable simultaneous observation of different physical parameters describing structural behaviour. For example, Abruzzi et al. developed an integrated monitoring system combining accelerometers, strain gauges, and numerical structural models, allowing measured data to be continuously compared with computational predictions and supporting the concept of intelligent structural monitoring [13]. Such systems significantly improve the quality of structural diagnostics but generally require sophisticated hardware configurations and centralized processing units, which increase implementation complexity and deployment costs.

Another important direction is represented by optical and photogrammetric monitoring techniques. Image-based approaches allow structural deformation and crack propagation to be evaluated without direct contact with the monitored object. One example is the monitoring system described in [14], where structural inclination is determined using a bubble level, a web camera, and an image-processing module integrated into a dedicated enclosure. A related photogrammetric approach combines camera systems with laser rangefinders to estimate crack geometry and monitor defect development during long-term observations [15]. Although these techniques provide accurate geometric measurements under controlled conditions, their practical application may be influenced by weather conditions, contamination of reference markers, varying illumination, and the computational complexity required for image processing.

Growing attention has also been paid to environmental monitoring systems that can complement conventional SHM platforms. Comprehensive reviews have demonstrated the rapid development of distributed sensing networks for measuring environmental parameters such as temperature, humidity, and air quality in smart-city applications [16]. Similar concepts have been implemented in low-cost wireless platforms integrating environmental sensors with intelligent building management systems [17]. While these solutions effectively support environmental monitoring, they generally do not incorporate structural sensing components, limiting their applicability to comprehensive diagnostics of engineering structures. The integration of environmental measurements with structural parameters therefore remains an important research direction.

Remote monitoring architectures based on browser/server technologies have also demonstrated promising capabilities for equipment diagnostics and long-term condition assessment. Centralized monitoring platforms simplify data storage, visualization, and remote analysis while supporting automated fault detection procedures [18]. However, their dependence on permanent server infrastructure and stable communication channels may reduce reliability under real operating conditions where network interruptions or communication delays cannot be avoided. Consequently, decentralized monitoring concepts capable of maintaining autonomous operation have attracted increasing research interest.

A comprehensive review of modern sensing technologies presented in [19] confirms that contemporary SHM systems employ a broad spectrum of sensing principles, including vibration, strain, displacement, acoustic, optical, magnetic, and environmental measurements. Although these technologies provide increasingly accurate information about structural condition, the review also demonstrates that communication architecture has received considerably less attention than sensing hardware itself. In particular, practical issues associated with wireless transmission reliability, synchronization between distributed sensing nodes, communication latency, and preservation of measurement quality under realistic operating conditions remain insufficiently investigated. These unresolved issues become especially important when standard wireless communication technologies are considered for long-term monitoring of large engineering structures.

Recent studies have proposed several distributed monitoring systems employing different wireless communication technologies and sensing platforms for structural and environmental monitoring.

Study [20] presents a wireless vibration monitoring node based on a MEMS accelerometer and a low-power microcontroller. The proposed platform enables vibration signal acquisition, local storage, and wireless data transmission. Its main advantage is the compact implementation of an autonomous sensing node. However, the study focuses on a single measurement unit and does not investigate distributed network organization, routing mechanisms, mathematical network modeling, or communication performance evaluation.

A monitoring system for historical masonry buildings based on LoRaWAN technology is proposed in [21]. The developed solution enables long-term monitoring of temperature, humidity, crack opening, and structural displacement while providing reliable long-range communication for cultural heritage applications. Nevertheless, the proposed architecture is based on LPWAN communication and does not address distributed Wi-Fi networking, routing algorithms, or analysis of wireless transmission quality.

An intelligent wireless monitoring system for residential buildings is described in [22]. The proposed architecture integrates distributed sensor nodes, cloud services, and mobile applications to support fire detection, gas leakage monitoring, and smart home management. Although the system employs a distributed wireless sensor network, it is primarily intended for smart home applications and does not support heterogeneous structural sensing modules required for monitoring displacement, crack propagation, or magnetic field variations.

A comprehensive review of wireless structural health monitoring systems for high-rise buildings is presented in [23]. The study summarizes recent developments in sensing technologies, wireless communication, and cloud-based monitoring platforms. However, it does not include the implementation or experimental validation of a unified distributed monitoring architecture.

An ESP32-based wireless environmental monitoring platform is presented in [24]. The proposed system integrates temperature, humidity, carbon dioxide, and occupancy sensing into a cloud-connected monitoring platform. Although the hardware architecture demonstrates good flexibility, the study does not include a mathematical communication model, a distributed transmission algorithm, or statistical evaluation of communication reliability using MAE and RMSE metrics.

Another distributed environmental monitoring platform based on ESP32 microcontroller (Espressif Systems, Shanghai, China) technology is described in [25]. The proposed solution addresses sensor node design, energy efficiency, scalability, and web-based visualization of environmental data. However, the architecture is not implemented as a fully distributed monitoring framework and does not consider heterogeneous structural sensing modules or quantitative evaluation of communication performance.

A comparative analysis of the representative monitoring systems discussed above is presented in Table 1.

As summarized in Table 1, most existing monitoring systems focus on specific monitoring tasks, including vibration analysis, environmental monitoring, or smart building management. Although wireless communication is widely adopted, only a limited number of studies combine heterogeneous sensing modules, distributed IEEE 802.11 Wi-Fi communication, mathematical network modeling, distributed transmission algorithms, and comprehensive statistical validation within a single monitoring framework. In contrast, the proposed system integrates these components into a unified distributed architecture and validates its performance through comparative wired and wireless experiments using MAE, RMSE, and time-series analysis. These differences clearly demonstrate the novelty and scientific contribution of the proposed monitoring approach.

The comparative analysis presented above demonstrates that existing wireless structural health monitoring systems have primarily focused on individual aspects of monitoring, including the development of standalone sensing nodes, the application of specific wireless communication technologies such as LoRaWAN or conventional wireless sensor networks, or environmental and smart building monitoring. Although these studies have significantly advanced distributed sensing technologies, only limited attention has been devoted to the development of an integrated monitoring framework combining heterogeneous sensing modules, distributed communication, network modeling, and quantitative evaluation of wireless transmission performance.

The proposed monitoring system addresses these limitations by integrating several complementary components into a unified distributed IEEE 802.11 Wi-Fi-based architecture. Unlike conventional WSN and IoT-based solutions, the proposed framework combines heterogeneous sensing devices, including ultrasonic distance sensors, inertial measurement units, magnetometers, and environmental sensors, within a mesh/ad hoc communication architecture. Furthermore, the proposed system incorporates a mathematical network model, a distributed communication algorithm, and comprehensive statistical validation based on MAE, RMSE, and time-series analysis for comparative evaluation of wired and wireless data transmission.

Therefore, the scientific contribution of this work extends beyond the implementation of an individual sensing platform or communication technology. The proposed system establishes a scalable distributed SHM framework that integrates communication architecture, heterogeneous sensing, theoretical network analysis, and experimental validation into a unified monitoring platform suitable for long-term monitoring of civil engineering structures.

A preliminary structural and functional framework of a distributed Wi-Fi monitoring system for bridge structures and buildings was introduced in [26]. That study established the hierarchical organization of the monitoring system, defined the interaction between sensing modules and communication components, proposed the functional architecture of the distributed network, and developed the initial operating algorithm for wireless data transmission. These results formed the architectural basis for the subsequent development of the proposed monitoring platform.

Further development of the communication subsystem was presented in [27], where a dual-processor Wi-Fi platform for remote temperature and humidity monitoring was implemented. That work focused on optimizing wireless communication, reducing power consumption through different operating modes of the ESP-based platform, validating real-time data transmission, and demonstrating the integration of environmental sensors into a unified embedded hardware–software system.

Building upon these developments, the present study integrates the previously proposed architectural principles and communication mechanisms into a unified distributed SHM framework. In contrast to the previous studies, the proposed system combines heterogeneous sensing devices, including ultrasonic distance sensors, inertial measurement units, magnetometers, and environmental sensors, within a distributed IEEE 802.11 Wi-Fi mesh/ad hoc architecture. Furthermore, the system incorporates a mathematical network model, a distributed communication algorithm, and a comprehensive experimental evaluation based on statistical performance indicators, including MAE, RMSE, and time-series analysis. Consequently, the present work represents the evolution of earlier architectural and hardware concepts into a scalable distributed monitoring platform capable of reliable structural health monitoring of civil engineering structures.

In recent years, low-cost embedded platforms have become an important research direction in structural and environmental monitoring owing to the rapid development of open-source electronics and wireless communication technologies. The widespread adoption of Arduino, ESP8266, ESP32, Raspberry Pi, and similar embedded platforms has significantly reduced the cost of monitoring systems while simplifying sensor integration, data acquisition, and remote communication. A comprehensive systematic review conducted by Mobaraki et al. analyzed nearly one hundred studies devoted to low-cost monitoring systems and demonstrated that open-source embedded platforms are increasingly employed for building performance assessment, indoor environmental monitoring, structural diagnostics, and energy management. The review further highlighted Wi-Fi and ZigBee as the most frequently adopted communication technologies because of their flexibility, compatibility with Internet-based services, and ease of deployment in distributed monitoring applications [28].

The feasibility of employing low-cost embedded systems for structural health monitoring has subsequently been confirmed through numerous experimental studies. Komarizadehasl et al. developed a wireless bridge monitoring system based on inexpensive MEMS accelerometers and embedded communication modules, demonstrating that reliable vibration measurements can be achieved at a fraction of the cost of conventional SHM equipment while maintaining sufficient accuracy for modal analysis and long-term bridge monitoring [29]. Similarly, Andò et al. proposed a dedicated embedded sensing platform for structural health monitoring that combines low-cost hardware with specialized signal-recognition algorithms. Their results showed that careful integration of embedded electronics and signal processing enables economically efficient SHM solutions without significantly compromising measurement quality [30].

Recent review studies further indicate that the role of low-cost sensing technologies has expanded beyond conventional vibration monitoring. Komary et al. summarized recent advances in low-cost structural assessment, emphasizing that modern embedded platforms can integrate multiple sensing modalities, including accelerometers, displacement sensors, environmental sensors, and wireless communication modules within a single monitoring architecture [31]. Likewise, Liu et al. reviewed recent progress in smart sensors for structural health monitoring and non-destructive evaluation, concluding that intelligent sensing systems increasingly combine embedded processing, wireless communication, and data analytics to improve monitoring accuracy and support autonomous infrastructure inspection [32]. These developments demonstrate that low-cost sensing platforms have evolved from simple educational hardware into mature engineering tools capable of supporting practical SHM applications.

The application of low-cost embedded platforms has also expanded to monitoring construction materials and concrete structures. Porras et al. presented an Arduino-based wireless monitoring system for measuring temperature and humidity during early-age concrete curing, demonstrating that inexpensive embedded hardware can provide continuous monitoring of thermal behaviour and support quality control during industrial concrete production [33]. This work illustrates the growing applicability of open-source embedded systems beyond traditional structural monitoring and confirms their potential for a broad range of civil engineering applications.

Despite these advances, most published studies primarily focus on sensor development, measurement accuracy, or specific monitoring applications. Considerably less attention has been devoted to the communication architecture itself, particularly the influence of Wi-Fi network organization, communication parameters, distributed data transmission, and wireless network topology on the reliability of structural health monitoring. Consequently, the development of distributed Wi-Fi-based monitoring systems that jointly address sensing, communication reliability, and scalable network organization remains an important research challenge and constitutes the main focus of the present study.

The communication technology selected for a distributed SHM system largely determines its scalability, reliability, and practical applicability. Several wireless standards have therefore been considered for structural monitoring applications, including Z-Wave, Bluetooth Low Energy (BLE), Thread, ZigBee, cellular communication technologies, and Wi-Fi [34]. Each of these technologies addresses specific monitoring requirements but also introduces certain technical limitations.

Mesh-based protocols such as Z-Wave, Thread, and ZigBee are designed primarily for low-power distributed networks. They provide good energy efficiency and support self-organizing network topologies; however, their practical implementation is often constrained by regional frequency regulations, interoperability issues between manufacturers, limited communication bandwidth, or susceptibility to interference within the 2.4 GHz spectrum [35,36,37,38]. Bluetooth Low Energy offers excellent hardware compatibility and very low power consumption, but its relatively limited communication range and network organization make it less suitable for large-scale monitoring systems requiring simultaneous operation of numerous distributed sensing nodes [36]. Cellular communication technologies, including GSM, UMTS, and LTE, provide extensive geographical coverage and high communication capacity; nevertheless, they require complex telecommunication infrastructure, increase deployment costs, and reduce system autonomy because communication depends on external network operators [39].

Compared with these technologies, Wi-Fi provides a different balance between communication performance and implementation complexity. As one of the most widely deployed wireless standards, IEEE 802.11 offers high transmission rates, sufficient communication range for most engineering facilities, compatibility with existing network infrastructure, and straightforward integration with cloud-based monitoring platforms. Although Wi-Fi consumes more energy than specialized low-power protocols, its available bandwidth exceeds the requirements of typical SHM applications and enables reliable transmission of heterogeneous measurement data without additional communication gateways [40]. Moreover, modern Wi-Fi devices support configurable power-saving mechanisms together with Carrier Sense Multiple Access with Collision Avoidance (CSMA/CA), improving communication stability in networks consisting of multiple autonomous sensor nodes [41].

The literature review indicates that substantial progress has been achieved in the development of sensing technologies and distributed monitoring systems. Nevertheless, several important challenges remain unresolved. Existing studies rarely investigate how communication parameters of IEEE 802.11 networks—including transmission power, channel bandwidth, communication range, and network topology—influence the reliability of structural monitoring. Distributed routing and communication algorithms specifically adapted to Wi-Fi-based SHM systems remain limited, while practical recommendations for configuring autonomous wireless monitoring networks for buildings and bridge structures are still insufficiently developed. In addition, most published studies evaluate sensing technologies independently of the communication architecture responsible for reliable data delivery.

To facilitate a systematic comparison of the wireless communication technologies considered for structural health monitoring applications, their principal technical characteristics are summarized in Table 2.

These observations reveal a clear research gap. Current investigations mainly concentrate either on sensing techniques or on wireless communication technologies separately, whereas relatively few studies consider them as components of a unified distributed monitoring system. Consequently, the influence of Wi-Fi network organization on the stability, accuracy, and reliability of structural monitoring has not yet been comprehensively investigated under practical operating conditions.

Motivated by these challenges, this study proposes a distributed structural health monitoring system based entirely on standard IEEE 802.11 communication. In contrast to conventional SHM architectures that rely on centralized servers or specialized low-power wireless protocols, the proposed system employs a self-organizing Wi-Fi network in which autonomous sensor nodes exchange information directly while supporting flexible deployment and straightforward network expansion. The architecture integrates distance, magnetometric, and environmental sensors within a unified monitoring platform and combines distributed communication with local data acquisition and processing.

The proposed approach differs from existing studies in several important aspects. First, the research focuses not only on sensing technologies but also on the organization of the wireless communication network itself. Second, the influence of Wi-Fi communication on monitoring performance is investigated through a comparative analysis of wired and wireless data acquisition under identical experimental conditions. Third, the study considers heterogeneous sensing modules within a single distributed architecture, allowing the stability and consistency of different measurement channels to be evaluated simultaneously. Rather than relying on proprietary communication solutions, the proposed system demonstrates how widely available IEEE 802.11 technology can be effectively adapted for long-term structural monitoring.

Experimental validation was performed using a distributed prototype consisting of multiple autonomous monitoring nodes and a central data collection unit. A series of 500 consecutive measurements was carried out to evaluate communication reliability, transmission stability, and measurement consistency under wired and wireless operating modes. The acquired data were analyzed using statistical performance metrics, including the mean absolute error (MAE), root mean square error (RMSE), and time-series comparison methods. Particular attention was devoted to assessing whether wireless transmission influences the temporal characteristics and accuracy of structural monitoring data.

The obtained results demonstrate that, when properly configured, standard Wi-Fi communication provides reliable data transmission without significant degradation of measurement quality. The proposed architecture offers a scalable and fault-tolerant monitoring solution that can be implemented using commercially available networking equipment without requiring specialized communication infrastructure. Owing to its modular design, the developed system can also be adapted to a broad range of monitoring applications, including environmental monitoring, smart buildings, industrial facilities, energy systems, and precision agriculture.

The main objective of this study is to develop and experimentally validate a distributed Wi-Fi-based system for monitoring the technical condition of engineering structures. To achieve this objective, the study addresses four main tasks: (i) the development of a structural and functional architecture for distributed monitoring; (ii) the design of a distributed data transmission algorithm for autonomous Wi-Fi networks; (iii) the implementation of a multi-sensor monitoring platform integrating structural and environmental sensing modules; and (iv) the experimental evaluation of system performance through statistical comparison of wired and wireless data acquisition.

The main contribution of this work is the development and experimental validation of a comprehensive distributed structural health monitoring (SHM) platform based on IEEE 802.11 Wi-Fi technology. Unlike existing studies that primarily focus on individual sensing devices, specific wireless communication technologies, or isolated monitoring tasks, the proposed approach integrates communication architecture, heterogeneous sensing, network modeling, distributed data transmission, and experimental validation into a unified monitoring framework.

The main scientific contributions of this study can be summarized as follows:

- The development of a scalable three-layer distributed SHM architecture based on IEEE 802.11 Wi-Fi mesh/ad hoc communication;
- The integration of heterogeneous sensing modules, including ultrasonic, inertial, magnetometer, temperature, and humidity sensors, within a unified monitoring platform;
- The development of a mathematical network model and a distributed communication algorithm for reliable wireless data exchange;
- Comprehensive experimental validation through comparative analysis of wired and wireless data transmission using MAE, RMSE, and time-series analysis;
- Demonstration of the feasibility of employing standard Wi-Fi infrastructure as the communication backbone for long-term distributed monitoring of civil engineering structures.

The remainder of this paper is organized as follows. Section 2 presents the proposed system architecture and network model of the distributed Wi-Fi-based structural health monitoring system. Section 3 describes the materials, experimental setup, and research methodology. Section 4 presents the experimental results together with their analysis and discussion. Finally, Section 5 summarizes the main findings of the study and outlines directions for future research.

## 2. System Architecture and Network Model

Analysis of modern wireless monitoring systems indicates that, in addition to meeting functional requirements for data acquisition and transmission, an effective structural health monitoring architecture should be organized hierarchically. Such an organization enables all hardware and software components to operate as an integrated system while preserving the independence of individual subsystems. A hierarchical architecture also simplifies system expansion, facilitates maintenance and replacement of individual components without interrupting the monitoring process, and allows the monitoring platform to be adapted to different engineering structures. At the same time, the sensing subsystem must provide reliable access to the monitored object, accurate acquisition of measurement data, and stable wireless communication with higher network levels.

Based on these considerations, the proposed architecture was designed according to four main principles: (i) integration of hardware and software components within all functional subsystems; (ii) adaptation of hardware components to the requirements of structural health monitoring; (iii) compliance with the requirements for functionality, fault tolerance, scalability, and long-term operational reliability; and (iv) provision of secure and reliable communication between distributed monitoring nodes. Following these principles, the proposed system adopts a hierarchical organization consisting of three functional levels: the sensor level, responsible for distributed data acquisition; the network level, providing wireless routing and data aggregation; and the processing level, performing centralized and partially distributed processing, analysis, and visualization of monitoring data.

The proposed monitoring system is based on a hierarchical distributed communication model consisting of sensing, networking, and processing layers. From a network perspective, the architecture combines the advantages of IEEE 802.11 communication with distributed mesh/ad hoc interaction between sensor nodes, enabling flexible deployment without dependence on permanent wired infrastructure.

The theoretical basis of the proposed network model relies on the principle of distributed data exchange, where each sensor node operates as an autonomous cyber-physical element capable of acquiring measurement data, performing preliminary processing, and transmitting information to neighboring network devices. Unlike conventional centralized SHM systems, where communication is concentrated around a single control unit, the proposed architecture distributes communication functions among multiple network elements. This organization reduces the influence of individual node failures and improves overall network availability.

The scalability of the network is achieved through modular expansion of sensor nodes without requiring modifications to the overall communication architecture. As additional sensing modules are deployed, the communication topology can be extended while preserving the same routing principles and data transmission mechanisms. Consequently, the computational complexity of network expansion depends primarily on local node integration rather than complete network reconfiguration, making the proposed architecture suitable for large-scale monitoring applications.

From the communication standpoint, the proposed network model is designed to maintain stable data transmission despite variations in network topology and traffic conditions. The combination of distributed routing, standard IP communication, and IEEE 802.11 networking enables reliable packet delivery while preserving compatibility with existing communication infrastructure. Furthermore, the use of commercially available Wi-Fi technology simplifies system deployment and reduces implementation costs compared with proprietary monitoring solutions.

The proposed theoretical model establishes the relationship between the structural organization of the monitoring system, the communication architecture, and the experimental validation presented in the following sections. The developed network model therefore provides the theoretical foundation for evaluating transmission reliability, scalability, and fault tolerance under distributed structural health monitoring conditions.

**Figure 1:**
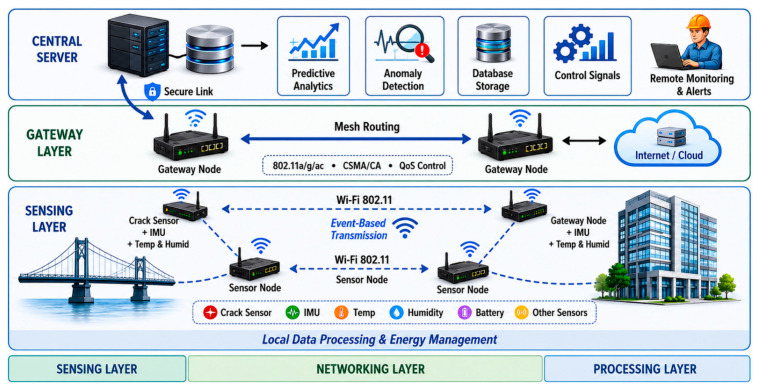
General architecture of the proposed distributed structural health monitoring system, illustrating the hierarchical organization of the sensing, communication, and processing layers and their interaction during data acquisition and transmission.

Figure 1 presents the general network architecture of the proposed monitoring system. The hierarchical organization separates sensing, communication, and data processing into independent functional layers while maintaining continuous interaction between them. Such an approach improves scalability, simplifies future expansion of the monitoring network, and increases overall system reliability by reducing dependencies between individual subsystems.

A key distinction between the proposed architecture and conventional wireless sensor networks is the use of IEEE 802.11 (Wi-Fi) communication instead of IEEE 802.15.4-based technologies. This choice provides several important advantages, including higher communication throughput, support for larger data packets, native compatibility with the IP protocol stack, and seamless integration with existing network infrastructure. These characteristics are particularly important for applications requiring reliable real-time transmission of heterogeneous monitoring data.

**Figure 2:**
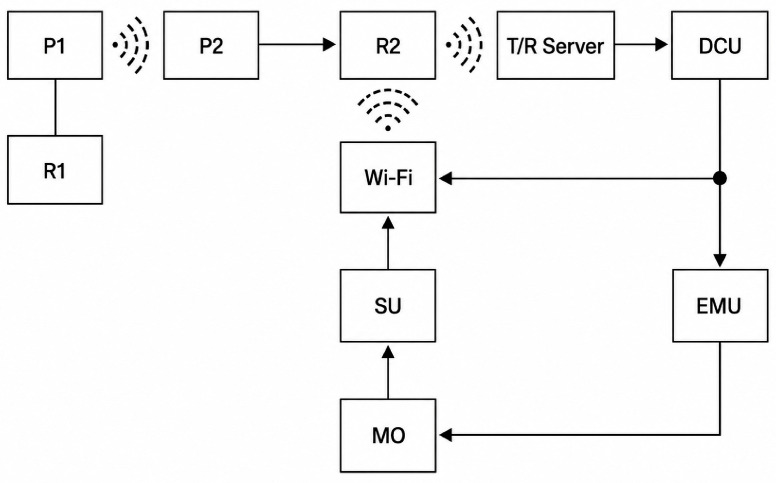
Structural diagram of the proposed distributed monitoring system showing the main hardware components, communication links, and data flow between sensor nodes, wireless network infrastructure, cloud server, and end-user interface. Abbreviations: R1—router on the transmitting side; P1—radio bridge on the transmitting side; P2—radio bridge on the receiving side; R2—router on the receiving side; T/R Server—transceiver server; DCU—data collection unit; Wi-Fi—Wi-Fi transceiver operating in client mode; SU—sensor unit; MO—monitored object; EMU—external monitoring interface.

Figure 2 illustrates the structural organization of the proposed distributed monitoring system and the interaction between its principal hardware components. The monitoring process begins with data acquisition at the sensor units installed on the monitored object. The collected information is then transmitted through the Wi-Fi communication subsystem and network infrastructure to the data collection and processing unit, where monitoring results are stored, analyzed, and made available to external monitoring interfaces. This organization separates sensing, communication, and data processing functions while maintaining reliable information exchange between all system components.

The data transmission process implemented in the proposed monitoring system begins at the sensor level, where measurement data describing the technical condition of the monitored object are continuously acquired. The monitored object corresponds to the engineering structure under investigation, while the sensor unit integrates several sensing modules responsible for measuring complementary structural and environmental parameters. These include an ultrasonic distance sensor for crack detection, temperature and humidity sensors for assessing environmental conditions, and an inertial measurement unit (IMU) incorporating an accelerometer, gyroscope, and magnetometer for monitoring spatial orientation and magnetic field variations.

The acquired measurements are transmitted to the Wi-Fi communication module operating in client mode. This module consists of an analog-to-digital converter (ADC), a microcontroller, and a wireless communication interface. Analog signals generated by the sensors, when applicable, are converted into digital form by the ADC before being processed by the microcontroller according to the embedded control algorithm. After local preprocessing, the measurement data are transmitted through the wireless interface to the transmitting-side router (R1), which establishes network connectivity with the remaining components of the monitoring system.

Reliable long-distance communication is achieved through a pair of radio bridges (P1 and P2). The transmitting radio bridge (P1) forwards the broadband data stream to the receiving bridge (P2), which subsequently transfers the information to the receiving-side router (R2). The measurement data are then delivered to the transceiver server (T/R Server), where they undergo additional processing before being forwarded to the data collection infrastructure. Depending on the operating scenario, the processed information may either be transmitted through the complete communication chain or provided directly to the external monitoring interface for real-time visualization and decision support. Finally, the processed measurement results are transferred via the HTTPS protocol to the data collection unit (DCU), which may be implemented using a personal computer, laptop, tablet, smartphone, or other embedded computing platform. The collected information is subsequently stored, analysed, and used to support assessment of the monitored structure and stabilization of the controlled process.

**Figure 3:**
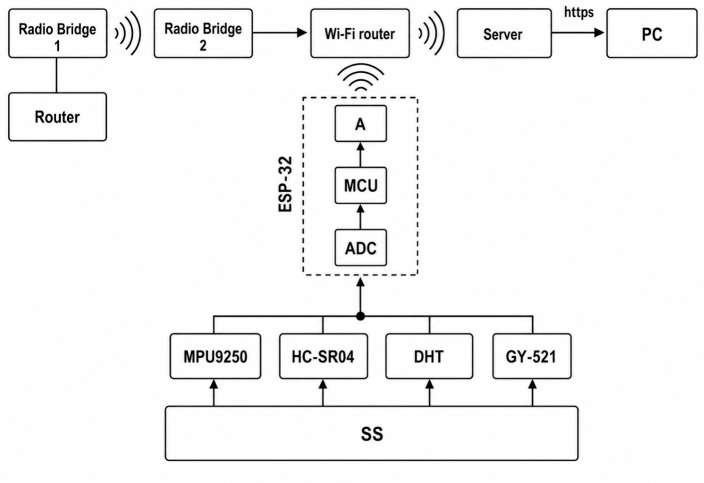
Functional diagram of the proposed distributed monitoring system illustrating the acquisition, processing, transmission, storage, and visualization of measurement data from heterogeneous sensing modules. Abbreviations: SS—signal source; HC-SR04—ultrasonic distance sensor; MPU-9250—inertial measurement unit (IMU) integrating an accelerometer, gyroscope, and magnetometer; GY-521—compact module incorporating a 3-axis accelerometer and a 3-axis gyroscope controlled via the I^2^C protocol; DHT—temperature and humidity sensor; ADC—analog-to-digital converter; MCU—microcontroller unit; A—antenna; ESP32—Wi-Fi module; ThingSpeak server—cloud platform used for data storage and visualization; Router—network device that forwards data packets between network segments according to routing rules; Radio Bridge 1—external wireless access point providing directional data transmission; Radio Bridge 2—network device that receives signals from another router or provider and distributes them within the local network; HTTPS—Hypertext Transfer Protocol Secure (default port 443); PC—personal computer.

Figure 3 summarizes the functional organization of the proposed distributed monitoring system and illustrates the complete workflow of measurement acquisition, wireless transmission, centralized processing, and visualization. Unlike the general architecture presented in Figure 2, the functional diagram emphasizes the interaction between individual hardware components and the sequence of information exchange throughout the monitoring process. This representation also demonstrates the integration of sensing devices, communication modules, networking equipment, cloud services, and user interfaces within a unified monitoring platform.

The structural and functional diagrams presented above describe the architecture and operational workflow of the proposed monitoring platform. However, reproducibility of the experimental study also requires a clear specification of the principal operating parameters of the developed system. Therefore, the main hardware configuration, communication settings, data acquisition strategy, and experimental conditions used throughout the validation process are summarized in Table 3.

As summarized in Table 3, the proposed monitoring platform combines commercially available communication equipment with low-power sensing nodes and a standard IEEE 802.11 Wi-Fi infrastructure. The selected operating parameters were chosen to ensure reliable wireless communication, stable data acquisition, and compatibility with heterogeneous sensing modules during all experimental scenarios. Providing these parameters facilitates reproducibility of the proposed monitoring system and enables future comparative studies under similar experimental conditions.

To validate the proposed monitoring architecture, an experimental study was conducted in which 500 consecutive measurements were acquired for each sensing channel. The system integrates several complementary sensors selected according to the monitored physical parameters. Crack development is monitored using the HC-SR04 (ELECFreaks Co., Shenzhen, China) ultrasonic distance sensor, which was selected because it provides accurate digital distance measurements based on the ultrasonic time-of-flight principle while remaining inexpensive and easily integrated with embedded microcontroller platforms. During operation, the sensor emits an ultrasonic pulse and determines the distance to the monitored surface by measuring the propagation time of the reflected signal, thereby enabling continuous observation of structural displacement and crack evolution.

Four sensors of this type were used in the experimental setup.

Four HC-SR04 ultrasonic sensors were installed at predefined locations on the monitored structure to record crack propagation and local structural displacement. Their non-contact operating principle and millimetre-level measurement resolution make them suitable for continuous laboratory monitoring while maintaining low implementation cost and straightforward integration with the ESP32 controller.

To monitor structural dynamics, the proposed system employs both MPU-6050 (GY-521) (Shenzhen, China) and MPU-9250 (TDK InvenSense Inc., San Jose, CA, USA) inertial measurement units. The MPU-6050 provides six-degree-of-freedom inertial measurements, whereas the MPU-9250 additionally incorporates a three-axis magnetometer, enabling estimation of absolute orientation and compensation of gyroscope drift. Sensor nodes equipped with these modules are distributed over the monitored structure according to its geometry and communicate with independent ESP32-based control units. To improve energy efficiency during long-term monitoring, the nodes operate in a periodic measurement mode using deep-sleep mechanisms, activating sensing and wireless communication only during scheduled acquisition cycles.

Environmental conditions are monitored using the DHT22 digital temperature and humidity sensor. Compared with the DHT11 (Aosong Electronics Co., Guangzhou, China), the DHT22 (Aosong Electronics Co., Guangzhou, China) provides a wider operating range together with higher measurement accuracy, making it more suitable for long-term structural monitoring under varying environmental conditions. The factory-calibrated digital output and simple single-wire interface further facilitate reliable integration into the proposed distributed monitoring platform.

The ESP32 serves as the central processing and communication module of each sensor node. In addition to executing local data acquisition and preprocessing algorithms, it provides integrated IEEE 802.11 wireless communication, eliminating the need for external communication hardware. Configurable power management functions allow periodic activation of sensing and communication tasks, reducing energy consumption while maintaining stable wireless connectivity. During operation, the firmware controls sensor polling, timer synchronization, wireless communication, and transmission of measurement data to the server for subsequent processing and visualization.

The proposed monitoring platform integrates sensing modules that provide complementary information about both the structural response and the surrounding environmental conditions. As summarized in Table 4, the selected hardware combines distance measurement, inertial sensing, magnetic field monitoring, and environmental measurements within a unified distributed architecture. This combination enables simultaneous observation of several factors affecting structural behaviour while maintaining a relatively simple and cost-effective hardware configuration.

All sensing devices provide digital communication interfaces fully compatible with the ESP32 platform, simplifying system integration and reducing communication overhead. The selected hardware also satisfies the principal design requirements formulated for the proposed monitoring system, including low implementation cost, modularity, scalability, and reliable operation under long-term monitoring conditions. The complementary characteristics of the selected sensors additionally reduce the dependence of structural assessment on a single measurement modality and improve the robustness of the monitoring process.

Wireless communication between the transmitting and receiving segments of the monitoring system is established through a standard IEEE 802.11 router, which provides sufficient communication range, support for multiple antenna configurations, and reliable operation under typical indoor monitoring conditions. The router serves as the central communication gateway, forwarding measurement data from distributed sensor nodes to the remote processing infrastructure. For data storage and processing, the proposed system employs the ThingSpeak cloud platform, which was selected for experimental validation because it provides a secure and readily available environment for rapid prototyping, remote data visualization, and long-term storage of measurement results. Although practical SHM deployments may utilize dedicated protected servers with restricted access, the selected cloud platform offers sufficient functionality for evaluating the proposed monitoring architecture under laboratory conditions.

Communication between the cloud server and the end-user device is implemented according to the OSI reference model using the HTTPS protocol, thereby ensuring secure transmission, data integrity, and confidentiality throughout the monitoring process. After data processing, the monitoring results become available to authorized users for visualization and further analysis through a standard web interface.

**Figure 4:**
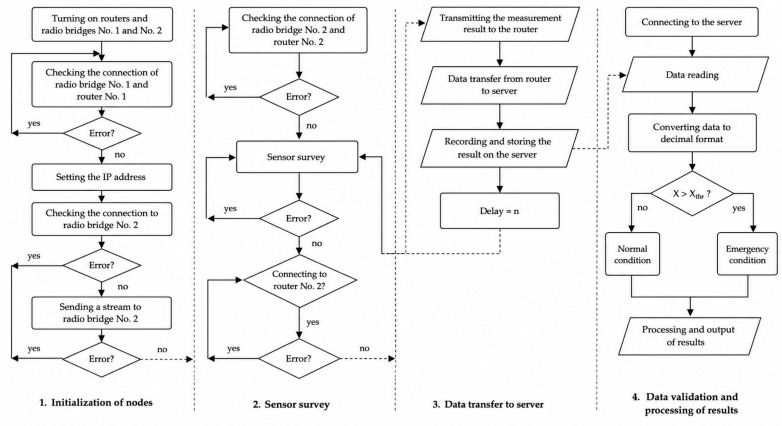
Workflow of the proposed distributed monitoring algorithm showing the sequence of sensor initialization, wireless communication, data transmission, server-side processing, and structural condition assessment. Solid arrows indicate the primary sequence of operations, whereas dashed arrows represent feedback loops, repeated operations, or transitions between successive processing stages.

Based on the proposed system architecture, a distributed monitoring algorithm was developed to coordinate periodic data acquisition, wireless communication, server-side processing, and user interaction. The workflow of the proposed algorithm is presented in Figure 4.

The algorithm is organized into four functional blocks representing the principal stages of information exchange within the monitoring system. The first block describes communication between the transmitting and receiving routers through the wireless bridge. The second block represents data transfer between the receiving bridge and the network router. The third block corresponds to the operation of the sensor node, including data acquisition, local preprocessing, and transmission of measurement results to the cloud server. Finally, the fourth block illustrates server-side processing together with visualization of monitoring results on the end-user device.

The monitoring cycle begins with initialization of the sensing modules and the ESP32 communication unit, followed by assignment of the network address and establishment of a wireless connection. Sensor measurements are then acquired periodically, and unsuccessful acquisition attempts automatically trigger repeated requests until valid measurement data are obtained. After successful acquisition, the sensor node establishes communication with the wireless router and transmits the collected information to the remote server. If the communication channel is temporarily unavailable, the transmission procedure is repeated until a stable connection is restored, thereby improving the reliability of wireless data delivery.

The connection between the cloud server and the end-user device is established using the HTTPS protocol, which provides encrypted communication, protects data integrity during transmission, and prevents unauthorized interception of monitoring information. User authentication supported by the cloud platform further restricts access to measurement data and system resources. Following each acquisition cycle, the sensor node enters a predefined low-power standby state before initiating the next measurement session. Structural condition assessment is subsequently performed by comparing the measured parameters with predefined threshold values. Measurements remaining within the acceptable range indicate normal operating conditions, whereas persistent exceedance of threshold values is interpreted as a potential indication of structural degradation requiring maintenance intervention. This decision-making strategy enables continuous monitoring of engineering structures while supporting early detection of abnormal behaviour and fault-tolerant operation of the distributed wireless monitoring network.

The network layer of the proposed distributed structural health monitoring system is responsible for node addressing, routing, communication management, and reliable data transmission within a dynamically changing wireless topology. Unlike conventional centralized SHM systems, which rely on hierarchical communication through a central server, the proposed architecture adopts a hybrid mesh/ad hoc communication model. This organization enables self-organization of sensor nodes, improves fault tolerance by supporting alternative communication paths, facilitates network expansion without major infrastructure modifications, and enhances the reliability of data delivery under changing network conditions.

A comparison of the proposed architecture with representative SHM communication approaches is presented in Table 5.

As shown in Table 5, the proposed architecture combines the scalability of cloud-based monitoring systems with the reliability of distributed wireless communication. Unlike conventional centralized solutions, the proposed approach distributes communication and processing tasks among multiple network elements, thereby reducing single points of failure while maintaining efficient data exchange between distributed sensing nodes.

To further evaluate the communication strategy, the proposed architecture was compared with several widely used MANET routing approaches. The results are summarized in Table 6.

Table 6 demonstrates that conventional MANET routing protocols were primarily developed for general mobile communication networks and only partially satisfy the requirements of structural health monitoring. In contrast, the proposed communication architecture incorporates energy-aware operation, quality-of-service support, and communication mechanisms specifically adapted to periodic acquisition of heterogeneous monitoring data, resulting in improved reliability and scalability for long-term SHM applications.

The proposed architecture was designed using a modular communication concept in which each additional sensor node operates independently while following the same communication and routing principles as existing nodes. Consequently, increasing the number of sensing devices does not require modifications to the overall network architecture but only local integration of new nodes into the existing communication infrastructure.

From the network perspective, the mesh/ad hoc organization improves scalability by allowing traffic to be distributed among multiple communication paths rather than relying on a single centralized gateway. As the monitored structure becomes larger and the number of sensing nodes increases, additional routers or relay devices can be incorporated to maintain communication quality while preserving the same distributed communication strategy.

Although the present study experimentally validates the proposed system using a limited number of sensing nodes, the developed network architecture is intended for significantly larger deployments, including bridges, industrial facilities, and multi-storey buildings. Future work will include large-scale experimental validation involving higher node densities and more complex engineering structures.

The communication hardware was selected according to the architectural requirements identified in the preceding analysis. To establish the backbone communication network, the experimental platform employs the MikroTik (MikroTik, Riga, Latvia) RBLHGG-60ad wireless bridge together with the MikroTik hAP ax3 router. These devices provide reliable long-distance communication, low transmission latency, and stable integration of geographically distributed Wi-Fi sensor nodes into a unified monitoring infrastructure.

The RBLHGG-60ad operates in the license-free 60 GHz frequency band and supports high-speed point-to-point communication between remote monitoring nodes. Operation within this frequency range substantially reduces interference from conventional 2.4 GHz and 5 GHz wireless networks while directional beamforming improves the signal-to-noise ratio and communication stability. Consequently, the backbone communication channel provides reliable transmission of heterogeneous monitoring data, including telemetry, vibration measurements, and environmental sensing information.

The MikroTik hAP ax3 functions as the edge gateway of the proposed monitoring system, aggregating traffic from distributed sensor nodes and coordinating communication with the central server. Support for IEEE 802.11ax communication together with local traffic management enables stable operation under varying network loads while maintaining low communication latency. In combination, the wireless bridge and aggregation router provide a scalable and fault-tolerant communication infrastructure capable of supporting continuous structural health monitoring under practical operating conditions.

Table 7 summarizes the functions of the principal communication devices employed in the proposed monitoring platform. Rather than serving as independent network components, these devices operate as an integrated communication infrastructure that supports reliable acquisition, transmission, processing, and visualization of structural monitoring data. The selected hardware combines local processing at the sensor node, high-speed backbone communication, intelligent traffic aggregation, and cloud-based data management, thereby providing a scalable and fault-tolerant platform suitable for long-term distributed structural health monitoring.

Although the primary objective of the proposed architecture is to ensure reliable distributed communication and data acquisition, cybersecurity remains an important consideration for practical deployment of structural health monitoring systems. In the proposed implementation, secure communication between the cloud platform and the end-user is provided through the HTTPS protocol, ensuring data confidentiality and integrity during transmission. Furthermore, the ThingSpeak platform employs authenticated user access to protect monitoring data from unauthorized use. For large-scale real-world deployments, additional security mechanisms such as mutual authentication of sensor nodes, secure cryptographic key management, encrypted firmware updates, role-based access control, and continuous network monitoring should be incorporated to further improve the resilience of the monitoring infrastructure against cyber threats. Although these mechanisms are beyond the scope of the present experimental study, they represent important directions for future development and practical implementation of distributed Wi-Fi-based structural health monitoring systems.

## 3. Materials and Methods

### 3.1. Experimental Setup

To evaluate the performance of the proposed distributed Wi-Fi-based structural health monitoring system, a series of laboratory experiments was conducted using the prototype described in the previous section. The objective of the experimental study was to assess the reliability of wireless data transmission, compare wired and wireless measurement acquisition, and evaluate the accuracy and stability of the proposed monitoring architecture under controlled operating conditions.

The experimental methodology consisted of four main stages. First, the distributed monitoring platform was assembled using the selected sensing modules, ESP32-based communication nodes, and the wireless network infrastructure described in the previous section. Second, repeated measurements were acquired simultaneously through both wired and wireless communication channels to enable direct comparison of transmission performance. Third, the collected datasets were preprocessed to eliminate invalid measurements and prepare the data for statistical analysis. Finally, the experimental results were evaluated using quantitative performance metrics and comparative time-series analysis to determine the influence of wireless communication on measurement accuracy and transmission reliability.

Unless otherwise stated, each experimental dataset consisted of 500 consecutive measurements acquired under identical laboratory conditions. The obtained data were subsequently analysed using statistical indicators, including the mean absolute error (MAE), root mean square error (RMSE), and graphical comparison of the recorded time series. These metrics were selected because they provide a quantitative assessment of agreement between wired and wireless measurements while allowing evaluation of communication stability and measurement consistency throughout the monitoring process.

### 3.2. Communication Delay Evaluation and Wireless Channel Characterization

Reliable operation of a distributed structural health monitoring (SHM) system depends not only on the accuracy of sensing devices but also on the characteristics of the wireless communication channel. Communication delay directly affects synchronization between distributed sensing nodes and the timely delivery of monitoring information, while the quality of the radio channel determines the stability and reliability of wireless data transmission. Therefore, in addition to evaluating the sensing performance, the communication characteristics of the proposed IEEE 802.11 Wi-Fi network were investigated experimentally and analytically.

For the experimental characterization of the wireless communication channel, an HSA870 spectrum analyzer (Hantek Electronic Co., Qingdao, China) was employed. The instrument was used to analyse the principal characteristics of the IEEE 802.11 radio interface, including carrier frequency, occupied bandwidth, channel power, adjacent-channel power, harmonic components, spectral distribution, and received signal strength. Owing to its wide range of measurement functions, the spectrum analyzer enables comprehensive evaluation of radio-frequency characteristics and provides an objective assessment of the communication quality of the developed distributed monitoring platform.

**Figure 5:**
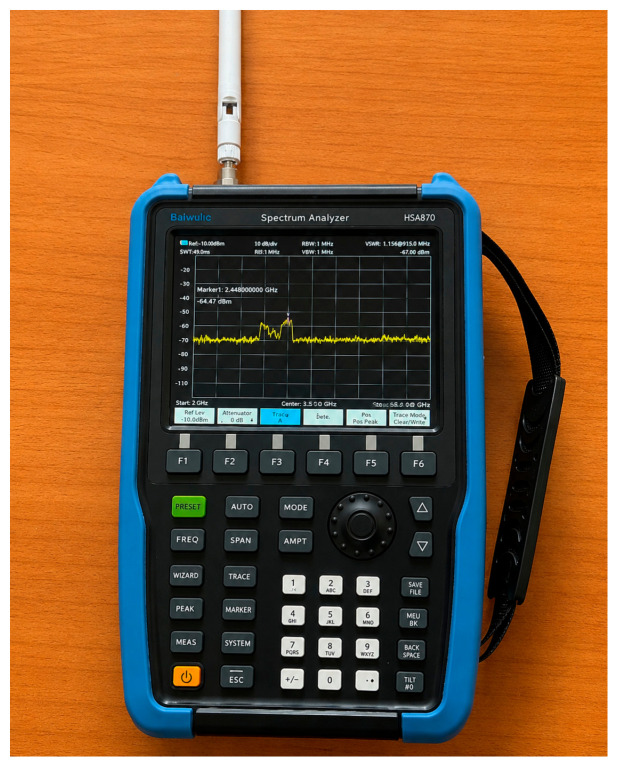
Experimental evaluation of the IEEE 802.11 wireless communication channel using the HSA870 spectrum analyzer.

Figure 5 presents the HSA870 spectrum analyzer during the experimental evaluation of the wireless communication channel.

The experimental measurements showed that the received signal strength varied from −38 dBm to −46 dBm within the operating frequency range of 2.1–2.5 GHz. Such signal levels correspond to favourable communication conditions and indicate a stable wireless connection with a high signal-to-noise ratio. Under these conditions, the communication channel provides low transmission latency, negligible packet loss, and sufficient throughput for continuous monitoring of engineering structures. It should be noted, however, that these characteristics correspond to the laboratory-scale experimental configuration. During large-scale deployment or when communication distances increase, additional investigations are required to evaluate the influence of propagation losses on network performance.

To complement the experimental measurements, a mathematical model describing wireless data transmission was developed in MATLAB/Simulink R2024a (MathWorks Inc., Natick, MA, USA). The objective of the model was to analyse the influence of communication delay and packet-loss probability on the stability of the wireless transmission process. Unlike the experimental measurements, which characterize the actual radio channel, the simulation model enables investigation of the communication behaviour under different operating conditions and network configurations.

The packet transmission process is described by the following nonlinear first-order differential equation:

(1) $${y_{si}}^{\prime }\left(t\right)+\alpha p\left(t\right)y^{2}\left(t\right)=\beta R^{-1}\left(1-p\left(t\right)\right)$$

where

- ${y_{si}}^{\prime }\left(t\right)$ is the packet transmission rate (packets/s);
- $p\left(t\right)$ is the packet-loss probability;
- α is the multiplicative decrease coefficient after packet loss;
- *β* is the additive increase coefficient during successful transmission;
- *R* is the communication delay.

For implementation in MATLAB/Simulink, Equation (1) was transformed into:

(2) $${y_{si}}^{\prime }\left(t\right)+Ay^{2}\left(t\right)=B$$

Accordingly, the transmission rate can be expressed as

(3) $${y_{si}}^{\prime }\left(t\right)=B-Ay^{2}\left(t\right)$$

**Figure 6:**
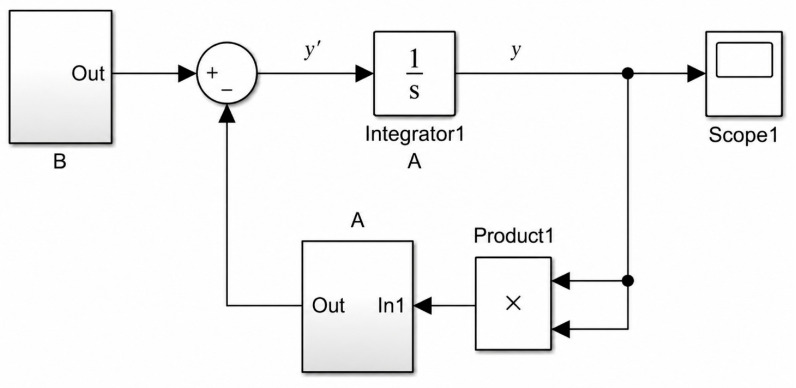
MATLAB/Simulink model of the proposed wireless communication channel. Here, A and B denote the coefficients defined by Equations (2) and (3), while the remaining symbols correspond to standard MATLAB/Simulink functional blocks.

The overall structure of the developed communication model is presented in Figure 6.

**Figure 7:**
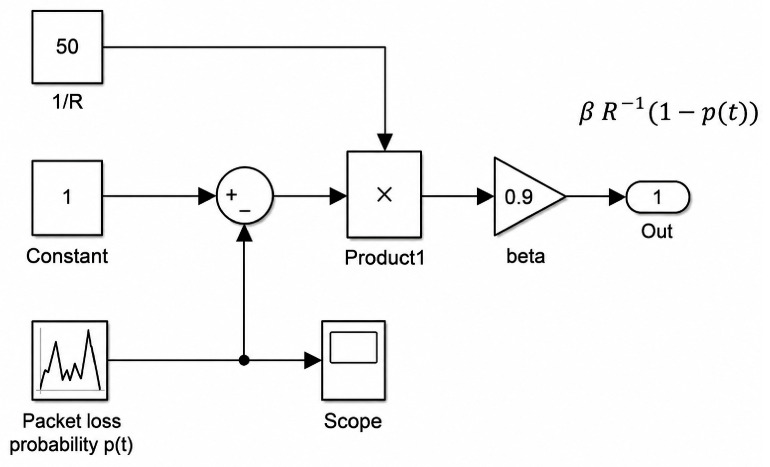
MATLAB/Simulink implementation of coefficient B.

**Figure 8:**
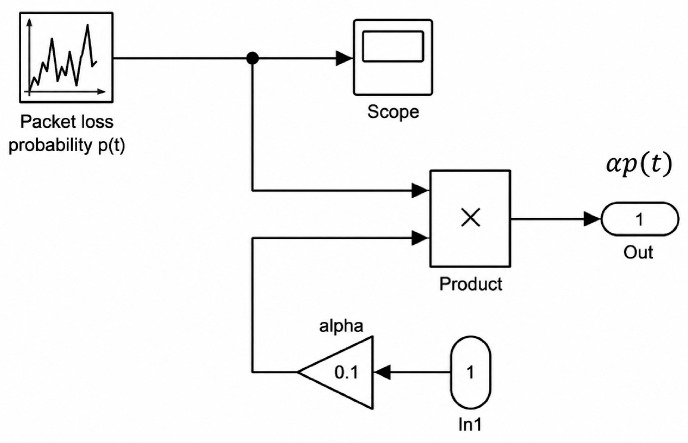
MATLAB/Simulink implementation of coefficient A.

For clarity, the coefficients A and B were implemented as independent functional blocks, illustrated in Figure 7 and Figure 8, respectively.

During the simulation, the average communication delay was set to 20 ms, corresponding to the experimentally observed communication conditions. Consequently, the reciprocal delay parameter was equal to R^−1^ = 50. The packet-loss probability was varied within the range of 0–0.1, representing typical operating conditions of an IEEE 802.11 Wi-Fi network with a stable communication link. The coefficients were selected as α = 0.1 and β = 0.9, providing a realistic representation of additive increase and multiplicative decrease mechanisms during packet transmission.

The developed communication model demonstrated stable transmission behaviour and confirmed the suitability of the selected wireless communication architecture for distributed structural health monitoring. The combination of experimental radio-frequency measurements and analytical modelling establishes a theoretical basis for the subsequent evaluation of packet loss, network throughput, and energy consumption presented in the following subsections.

### 3.3. Packet Loss Analysis

Reliable packet delivery is one of the key requirements for distributed structural health monitoring systems because missing measurements may lead to incorrect interpretation of the structural condition. Therefore, in addition to evaluating communication delay, the packet transmission reliability of the proposed wireless monitoring platform was investigated experimentally and verified using numerical simulation.

During the experimental validation, each sensing channel generated 500 consecutive measurements, which were transmitted through the complete communication chain consisting of the ESP32 sensing node, Wi-Fi network, router, cloud server, and monitoring workstation. Throughout the experimental campaign, no missing values were detected in any of the recorded datasets, indicating that all transmitted measurement packets were successfully received and stored. Consequently, under the investigated laboratory conditions, the observed packet loss rate was approximately 0%.

Although the experimental results demonstrated reliable packet delivery, communication performance under different operating conditions may vary depending on transmission distance, interference level, and radio propagation conditions. Therefore, an additional numerical investigation was carried out using the MATLAB/Simulink model introduced in the previous subsection.

Within the communication model, the packet-loss probability p(t) was treated as a variable parameter describing the stochastic behaviour of the wireless channel. The probability was varied within the interval 0–0.1, corresponding to communication conditions ranging from ideal transmission to moderate packet degradation typically encountered in IEEE 802.11 wireless networks.

**Figure 9:**
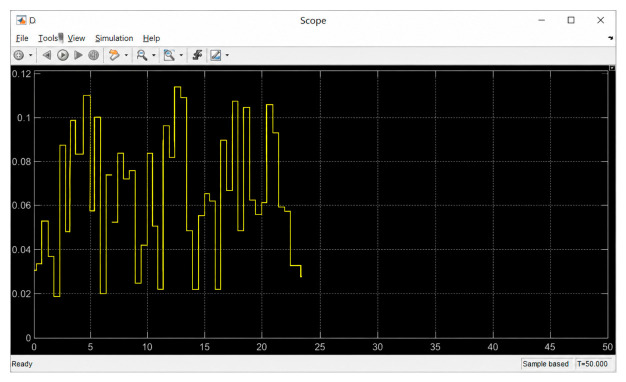
Packet-loss model used for simulation of wireless communication.

Figure 9 illustrates the packet-loss generator implemented in MATLAB/Simulink.

**Figure 10:**
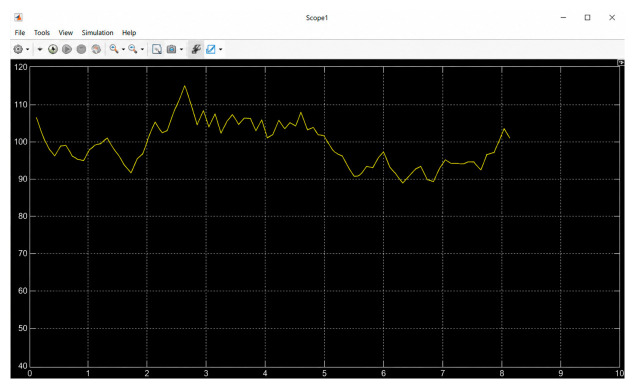
Simulated packet transmission under varying packet-loss conditions.

The generated packet-loss sequence was subsequently introduced into the transmission model to evaluate its influence on communication stability. The corresponding simulated transmission behaviour is presented in Figure 10.

The simulation results indicate that the proposed communication architecture remains stable under low packet-loss conditions and preserves continuous information exchange between distributed sensing nodes. The experimentally observed absence of missing measurements is consistent with the simulation results for low values of p(t), confirming the adequacy of the developed mathematical model.

The combined experimental and numerical analysis demonstrates that the proposed Wi-Fi-based monitoring system provides reliable packet delivery under the investigated operating conditions. At the same time, the simulation framework enables further investigation of communication reliability under more demanding scenarios, including increased transmission distances, higher interference levels, and larger-scale sensor deployments, which will be addressed in future work.

### 3.4. Throughput Assessment

The throughput of the wireless communication channel is one of the principal indicators determining the capability of a distributed monitoring system to transmit measurement data continuously without introducing communication bottlenecks. Sufficient throughput is particularly important in structural health monitoring applications involving simultaneous acquisition of heterogeneous sensor data and real-time information exchange between distributed sensing nodes.

The communication throughput of the proposed monitoring system was evaluated using the radio-frequency measurements described in the previous subsection. Based on the HSA870 spectrum analyzer measurements, the operating characteristics of the IEEE 802.11 Wi-Fi communication channel were analysed, including signal strength, occupied bandwidth, and channel quality. These parameters provide an indirect assessment of the achievable transmission performance under the investigated operating conditions.

The measured received signal strength ranged from −38 dBm to −46 dBm, indicating a stable communication link throughout the experimental campaign. Within this signal range, the wireless network maintained uninterrupted communication between the sensing nodes, network infrastructure, and cloud server. No communication interruptions or transmission failures were observed during the acquisition of the complete experimental datasets.

The developed monitoring platform was designed for periodic transmission of relatively small telemetry packets containing distance, magnetic field, temperature, and humidity measurements. Consequently, the communication load generated by the sensing nodes remained considerably below the available bandwidth of the IEEE 802.11 wireless channel. Under these operating conditions, the available throughput was sufficient to support continuous acquisition and transmission of measurement data without introducing communication delays associated with network congestion.

Although the present study primarily focused on evaluating the reliability of distributed data transmission rather than determining the theoretical maximum throughput of the IEEE 802.11 standard, the experimental results confirm that the selected wireless infrastructure provides adequate communication capacity for the proposed structural health monitoring application. The obtained results also indicate that the available communication resources allow future expansion of the monitoring platform through the integration of additional sensing nodes and heterogeneous sensor modules.

The throughput analysis therefore confirms that the proposed Wi-Fi-based communication architecture satisfies the transmission requirements of the developed distributed monitoring system while preserving stable data exchange throughout the experimental validation.

### 3.5. Energy Consumption Optimization

Power consumption is an important design consideration for distributed structural health monitoring systems, particularly when sensor nodes are expected to operate autonomously for extended periods or are deployed in locations where continuous power supply is unavailable. Consequently, the proposed monitoring platform was designed with particular attention to reducing energy consumption while maintaining reliable wireless communication.

The developed monitoring system employs the NodeMCU ESP8266 (AI-Thinker Technology Co., Shenzhen, China) wireless communication module, which provides several operating modes with different power requirements. These include the Active Mode, Modem Sleep Mode, Light Sleep Mode, and Deep Sleep Mode. Each operating mode offers a different compromise between communication availability and energy efficiency, allowing the system configuration to be adapted according to monitoring requirements.

The principal characteristics of the available power-saving modes of the ESP8266 module are summarized in Table 8.

Among the available operating modes, the Deep Sleep Mode was selected for the proposed monitoring platform because it provides the lowest energy consumption while preserving periodic wireless data transmission. During this operating mode, only the real-time clock (RTC) remains active, whereas the processor, wireless transceiver, and peripheral modules are temporarily disabled. The sensing node automatically wakes up after the predefined sleep interval, performs sensor measurements, transmits the acquired data to the cloud server, and subsequently returns to the low-power state.

The operating sequence of the sensing node consists of four consecutive stages:

Establishing a wireless connection with the IEEE 802.11 access point;

- Acquiring measurements from the connected sensing modules and transmitting the collected data using the MQTT communication protocol;
- Entering the Deep Sleep Mode for a predefined time interval specified in microseconds;
- Automatically repeating the measurement and transmission cycle after wake-up.

According to the ESP8266 hardware specifications, the Deep Sleep operating mode reduces the average current consumption to the microampere range, whereas significantly higher current values are observed during the active communication modes. Consequently, the proposed operating strategy substantially decreases the overall energy demand of the monitoring platform and increases the potential operating lifetime of autonomous sensing nodes.

It should be emphasized that the objective of the present study was not to experimentally determine the absolute energy consumption of the monitoring platform but rather to implement an energy-efficient communication strategy suitable for distributed structural health monitoring applications. The selected Deep Sleep operating mode provides an effective compromise between communication availability and power consumption while preserving reliable acquisition and transmission of monitoring data.

The proposed energy management strategy also provides a foundation for future large-scale deployment of the monitoring system. As the number of distributed sensing nodes increases, optimization of duty cycles, adaptive wake-up scheduling, and intelligent power management algorithms can further reduce the overall energy demand while maintaining continuous structural monitoring.

The communication performance evaluation demonstrates that the proposed distributed Wi-Fi monitoring platform provides reliable operation from both communication and energy-efficiency perspectives. The combination of experimental radio-frequency measurements, mathematical modelling, packet-loss analysis, throughput assessment, and low-power operating strategy confirms the suitability of the selected IEEE 802.11 communication architecture for distributed structural health monitoring applications. These results establish the communication reliability of the proposed monitoring platform and provide the methodological basis for analysing the measurement accuracy and transmission consistency presented in the following section. Based on the validated communication infrastructure, the subsequent experimental study focuses on the comparative evaluation of wired and wireless measurements acquired from the integrated sensing modules under representative operating conditions.

## 4. Results and Discussion

Experimental validation of the proposed distributed monitoring system was performed using the architecture and methodology described in the previous sections. The primary objectives of the experiments were to evaluate the reliability of wireless data transmission, assess the consistency between wired and wireless measurement acquisition, and quantify the influence of the communication channel on measurement accuracy. For this purpose, a series of 500 consecutive measurements was acquired for each sensing channel, including distance sensors, inertial measurement units, and environmental sensors.

The collected datasets were analysed using statistical methods and comparative time-series evaluation. The analysis included calculation of the mean absolute error (MAE) and root mean square error (RMSE), together with graphical comparison of wired and wireless measurements. These statistical indicators were selected to quantify the agreement between both acquisition methods and to identify possible communication-induced deviations. Throughout the experimental campaign, no missing samples or communication interruptions were detected, indicating stable operation of both the wireless transmission channel and the proposed distributed monitoring architecture.

**Figure 11:**
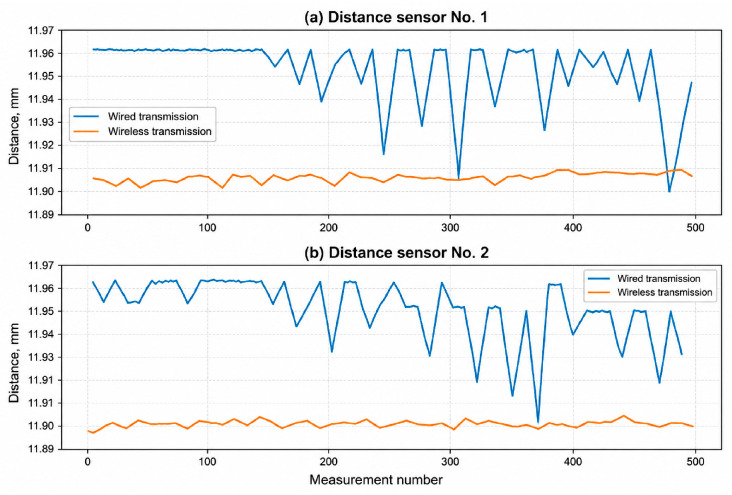
Comparison of wired and wireless measurements obtained from two HC-SR04 distance sensors during 500 consecutive measurements: (**a**) distance sensor No. 1; (**b**) distance sensor No. 2.

Figure 11 compares the measurements obtained from two HC-SR04 distance sensors using wired and wireless communication channels. The figure demonstrates that both acquisition methods exhibit nearly identical temporal behaviour throughout the complete measurement sequence, indicating that wireless transmission preserves the dynamics of structural displacement without introducing observable distortions.

Quantitative analysis confirms the close agreement between the two acquisition methods. For distance sensor No. 1, the mean absolute error (MAE) was approximately 0.043 mm, while the root mean square error (RMSE) reached 0.045 mm. Corresponding values for distance sensor No. 2 were 0.047 mm and 0.049 mm, respectively. No abrupt fluctuations, communication failures, or abnormal deviations were observed throughout the 500 consecutive measurements, and both sensors maintained stable waveform characteristics during the entire observation period.

The obtained results demonstrate that the proposed Wi-Fi-based communication architecture introduces only negligible transmission errors while preserving the temporal characteristics of the measured signals. This level of agreement confirms the suitability of the proposed distributed monitoring system for applications requiring precise detection of crack propagation and small structural displacements, where reliable preservation of measurement dynamics is essential.

**Figure 12:**
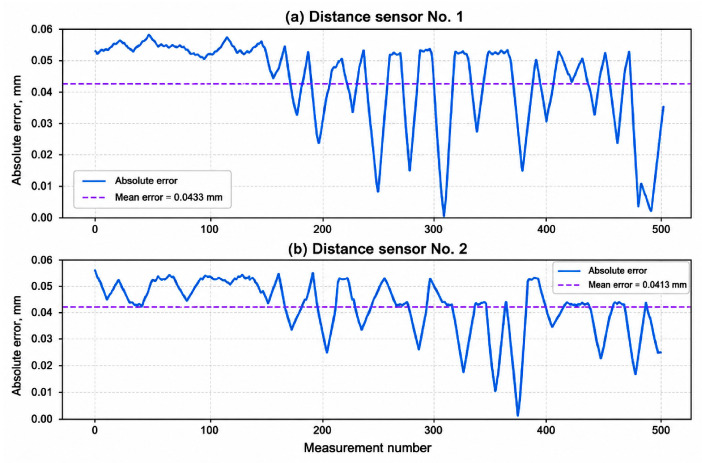
Absolute measurement error of wireless distance measurements relative to the corresponding wired measurements for two HC-SR04 distance sensors over 500 consecutive measurements: (**a**) distance sensor No. 1; (**b**) distance sensor No. 2.

Figure 12 presents the absolute error of wireless distance measurements with respect to the corresponding wired measurements for both HC-SR04 sensors. The error distributions remain stable throughout the complete sequence of 500 measurements and show no evidence of cumulative communication errors or progressive measurement drift.

Quantitative evaluation demonstrates that the MAE for distance sensor No. 1 was approximately 0.043 mm, while the maximum observed deviation did not exceed 0.057 mm. For distance sensor No. 2, the corresponding MAE was approximately 0.047 mm, with a maximum deviation of 0.062 mm. Although small local fluctuations are visible in both datasets, their amplitudes remain nearly constant throughout the experiment and do not develop into systematic measurement bias.

The consistently low error values confirm that the proposed wireless communication architecture preserves measurement accuracy during long-term operation. These results further support the applicability of the developed system for continuous crack monitoring and displacement measurements, where stable transmission of small geometric changes is essential.

**Figure 13:**
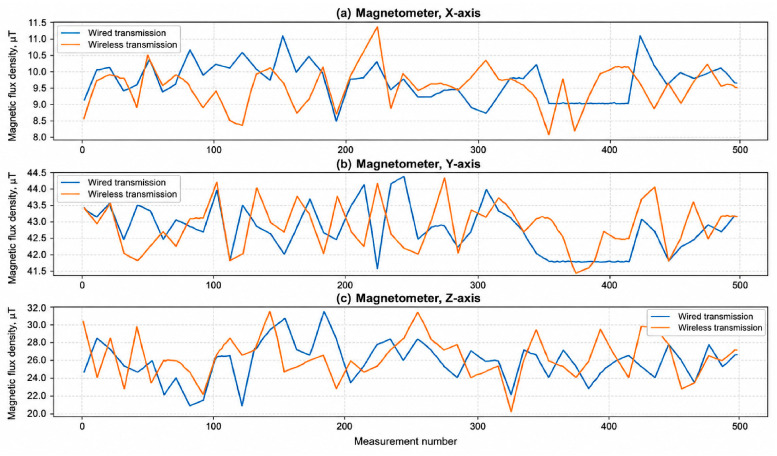
Comparison of wired and wireless magnetometer measurements obtained from the MPU-9250 sensor during 500 consecutive measurements: (**a**) X-axis; (**b**) Y-axis; (**c**) Z-axis.

Figure 13 compares wired and wireless magnetometer measurements acquired along the three orthogonal coordinate axes. In contrast to the distance sensors, the magnetometer signals exhibit higher sensitivity to minor transmission-induced fluctuations owing to the increased sensitivity of magnetic field measurements. Nevertheless, the overall temporal behaviour of the recorded signals remains highly consistent for both acquisition methods.

Among the three measurement axes, the Y-axis demonstrates the closest agreement between wired and wireless measurements, with an MAE of approximately 0.497 µT and an RMSE of approximately 0.639 µT. The corresponding values for the X-axis are 0.592 µT (MAE) and 0.769 µT (RMSE), whereas the Z-axis exhibits intermediate performance with an MAE of 0.556 µT and an RMSE of 0.680 µT. Despite the presence of localized deviations, all three channels preserve the principal waveform characteristics and accurately reproduce the temporal evolution of the magnetic field.

**Figure 14:**
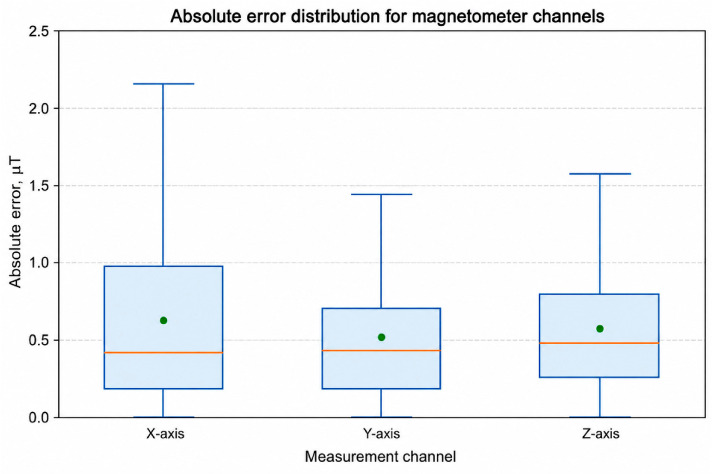
Distribution of absolute measurement errors for the magnetometer channels along the X-, Y-, and Z-axes. Outliers are omitted to improve visualization of the principal error distributions. The orange line represents the median, while the green dot denotes the mean value.

To further evaluate communication stability, Figure 14 presents the distribution of absolute measurement errors for the three magnetometer channels.

The error distributions confirm the observations obtained from the time-series analysis. The Y-axis exhibits the smallest dispersion of measurement errors, whereas the X-axis demonstrates the highest variability. Maximum deviations of approximately 2.330 µT, 2.165 µT, and 1.734 µT were observed along the X-, Y-, and Z-axes, respectively. Although magnetometer measurements are more susceptible to communication-related fluctuations than distance measurements, the obtained errors remain sufficiently small to preserve the dynamic characteristics of the measured magnetic field. Consequently, the proposed wireless monitoring system provides adequate performance for structural health monitoring tasks involving spatial orientation and magnetic field analysis.

**Figure 15:**
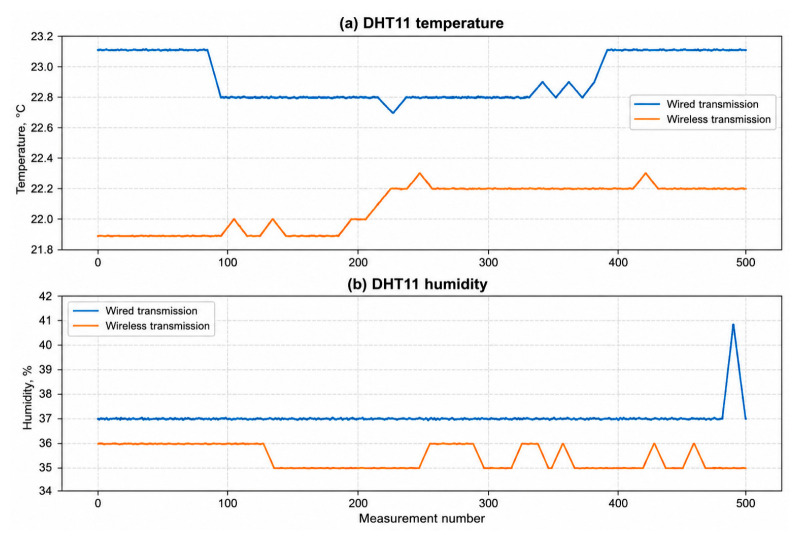
Comparison of wired and wireless measurements acquired using the DHT11 sensor during 500 consecutive measurements: (**a**) temperature; (**b**) relative humidity.

Following the evaluation of distance and magnetometer measurements, the proposed monitoring system was further assessed using environmental sensing channels. Figure 15 compares temperature and humidity measurements acquired with the DHT11 sensor through wired and wireless communication. Although both acquisition methods reproduce similar temporal trends, the agreement between the corresponding datasets is lower than that observed for the distance and magnetometer channels.

Quantitative analysis indicates that the temperature channel achieved an MAE of approximately 0.842 °C and an RMSE of 0.867 °C. The wireless measurements preserve the overall temperature profile; however, a slight systematic underestimation is observed throughout the experiment. The humidity channel exhibits greater variability, with an MAE of approximately 1.620%, an RMSE of 1.763%, and maximum deviations approaching 5.9%. Despite these differences, the wireless communication channel successfully preserves the general behaviour of both environmental parameters, confirming its suitability for environmental monitoring where moderate deviations are acceptable.

**Figure 16:**
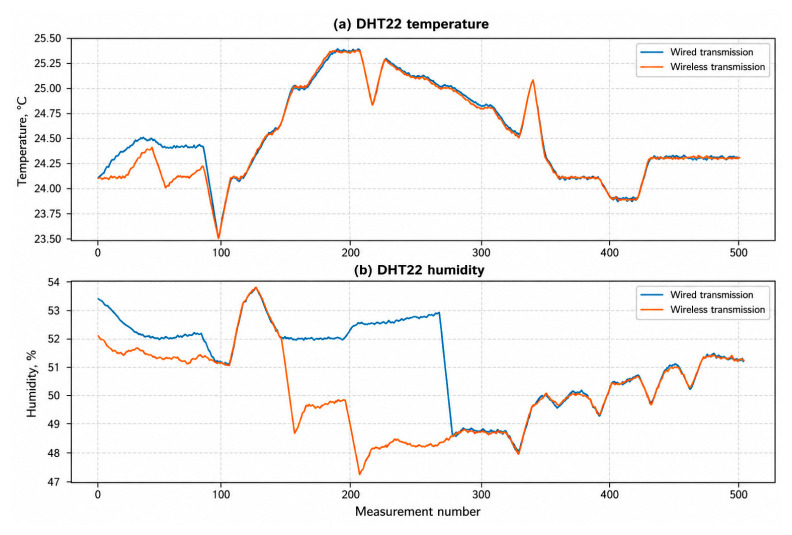
Comparison of wired and wireless measurements acquired using the DHT22 sensor during 500 consecutive measurements: (**a**) temperature; (**b**) relative humidity.

To investigate the influence of sensor accuracy on communication performance, the same experiments were repeated using the DHT22 sensor. The corresponding results are presented in Figure 16.

The DHT22 demonstrates substantially better agreement between wired and wireless measurements than the DHT11, particularly for temperature monitoring. The temperature channel achieved an MAE of approximately 0.048 °C and an RMSE of 0.104 °C, representing one of the highest levels of agreement observed during the entire experimental study. Although the humidity channel still exhibits increased variability (MAE ≈ 1.191%, RMSE ≈ 2.104%), the temporal evolution of humidity remains well preserved. These results indicate that measurement deviations are influenced primarily by the sensing characteristics of the environmental sensors rather than by the wireless communication channel itself.

It should be noted that the wired and wireless measurements were obtained using two identical DHT22 sensor modules operating simultaneously, with one connected through a wired interface and the other transmitting data via the proposed Wi-Fi communication architecture. Therefore, the comparison evaluates the capability of the wireless monitoring node to reproduce the measurements of the wired reference under identical operating conditions rather than assessing the communication channel in isolation.

The high level of agreement observed for the DHT22 temperature channel can be explained by several factors. First, the DHT22 is a factory-calibrated digital sensor providing stable digital output with high intrinsic accuracy, resulting in only minor inter-sensor deviations. Second, temperature changes relatively slowly and remains spatially uniform over short distances under laboratory conditions, allowing both sensor modules to record nearly identical temperature profiles throughout the experiment. In addition, no missing packets were detected during the transmission of the 500 consecutive measurements, indicating stable wireless communication during data acquisition.

Consequently, the observed Mean Absolute Error (MAE) of approximately 0.048 °C mainly reflects the intrinsic variability between two independent sensor modules rather than errors introduced by wireless transmission. The slightly higher Root Mean Square Error (RMSE) of approximately 0.104 °C is primarily associated with several short-duration local deviations between the two measurement series. These results demonstrate that the proposed Wi-Fi communication architecture preserves the integrity of temperature measurements and accurately reproduces the readings acquired through the wired reference channel.

**Figure 17:**
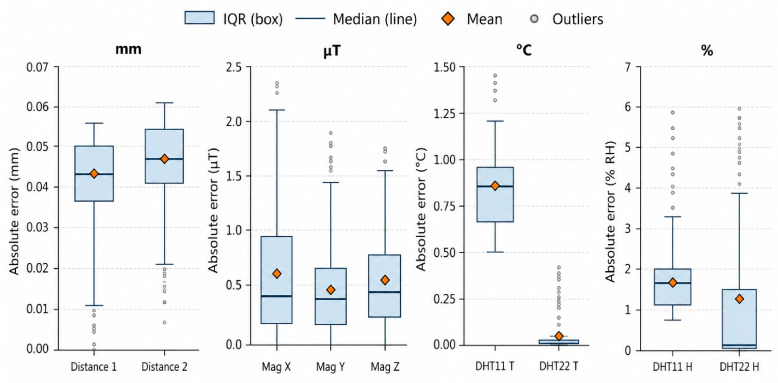
Distribution of the absolute error for all measurement channels of the developed monitoring system, grouped by physical unit (mm, µT, °C, and % RH); boxes denote the interquartile range and the median, diamonds the mean, and grey points the outliers.

**Figure 18:**
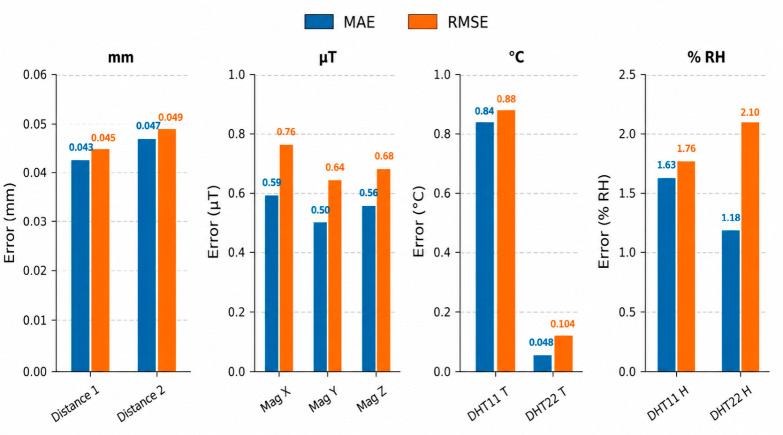
A brief comparison of the mean absolute error (MAE) and the root mean square error (RMSE) for all measurement channels of the developed monitoring system, grouped by physical unit (mm, µT, °C, and % RH).

To provide an integrated evaluation of all sensing channels, Figure 17 and Figure 18 summarize the absolute error distributions together with the corresponding statistical error metrics.

As shown in Figure 17, the integrated comparison of absolute errors across all measurement channels provides a comprehensive overview of the error distribution within the proposed distributed monitoring system. Since the analysed channels correspond to different physical quantities, they are grouped into four panels according to their measurement units (mm, µT, °C, and % RH), each with an independent vertical scale. This arrangement enables statistically meaningful comparison between channels measuring the same physical quantity while preserving an overall view of the measurement performance of the complete monitoring platform.

Among all sensing channels, the ultrasonic distance sensors demonstrate the highest measurement consistency. Distance sensor No. 1 achieves the lowest Mean Absolute Error (MAE) of approximately 0.043 mm, while Distance sensor No. 2 exhibits a slightly higher value of approximately 0.047 mm. Although the median error of Distance sensor No. 2 is marginally larger, both sensors are characterized by narrow interquartile ranges and a limited number of outliers, indicating highly stable measurement performance and excellent agreement between wired and wireless data transmission.

The magnetometer channels exhibit moderate error dispersion. The Y-axis demonstrates the smallest variability among the three magnetic field components, whereas the X-axis presents a wider interquartile range and several larger deviations. Nevertheless, the relatively small separation between the median and mean values indicates that the observed differences remain statistically stable and are not associated with systematic communication errors.

A markedly different behaviour is observed for the temperature and humidity measurements. The DHT22 temperature channel exhibits the best overall performance among the environmental sensors, with an MAE of approximately 0.048 °C. The corresponding boxplot is concentrated close to zero, while the median and mean almost coincide, indicating that the wireless transmission preserves the temperature measurements with negligible additional uncertainty. In contrast, the DHT11 temperature channel exhibits substantially larger dispersion, reflecting the lower measurement stability of this sensor.

The largest variability is observed for the humidity measurements. Although the DHT22 humidity channel exhibits a median value close to zero, numerous high-value outliers extending to approximately 5–6% RH indicate the presence of occasional large deviations. A similar behaviour is observed for the DHT11 humidity channel, although its interquartile range is noticeably wider. This behaviour is primarily attributed to the intrinsic characteristics of low-cost humidity sensing, including slower sensor response, higher sensitivity to local environmental fluctuations, and calibration differences between independent sensing modules, rather than to the wireless communication process itself. Since identical communication conditions were maintained for all sensing channels, the significantly lower error levels observed for the distance sensors and the DHT22 temperature channel confirm that wireless transmission is not the dominant source of measurement uncertainty.

From a statistical perspective, Figure 17 demonstrates that the proposed Wi-Fi-based monitoring architecture preserves measurement consistency across heterogeneous sensing channels. For most parameters, the interquartile ranges remain relatively narrow, while the mean values are located close to the corresponding medians, indicating the absence of systematic bias introduced by wireless communication. Consequently, the remaining discrepancies between the wired and wireless measurements are governed primarily by the metrological characteristics of the sensing modules themselves rather than by the communication subsystem. This distribution-based analysis complements the aggregated performance comparison presented in Figure 18, where the average error metrics are evaluated across all measurement channels.

The quantitative error metrics presented in Figure 18 provide a complementary assessment of the wireless transmission performance by comparing the Mean Absolute Error (MAE) and Root Mean Square Error (RMSE) for all sensing channels. Since the evaluated parameters are expressed in different physical units, the results are grouped into separate panels (mm, µT, °C, and % RH), each with an independent vertical axis. This arrangement enables direct comparison of MAE and RMSE within each measurement category while avoiding misleading comparisons between quantities expressed in different units.

The ultrasonic distance sensors exhibit the lowest MAE and RMSE values among all investigated channels, confirming the excellent agreement between the wired and wireless measurements during displacement monitoring. The very small difference between MAE and RMSE indicates that measurement errors remain consistently low throughout the experiment, without pronounced deviations. Similarly, the DHT22 temperature channel demonstrates outstanding performance, substantially outperforming the DHT11 temperature sensor in terms of both error metrics.

The magnetometer channels exhibit intermediate error levels. Although the absolute errors are higher than those observed for the distance sensors, the relatively small difference between MAE and RMSE indicates stable measurement performance and confirms that the wireless communication system preserves the consistency of the transmitted magnetic field measurements.

A different behaviour is observed for the humidity channels. Both DHT11 and DHT22 humidity measurements exhibit noticeably larger RMSE values than their corresponding MAE values, indicating that, while the majority of measurements remain close to the reference values, a limited number of observations contribute disproportionately to the squared-error metric. This behaviour is consistent with the outliers observed in Figure 11 and reflects the intrinsic characteristics of humidity sensing rather than systematic errors introduced by the wireless communication architecture.

From a technical perspective, the combined interpretation of MAE and RMSE provides considerably more information than either metric alone. When the two metrics are similar, the error distribution is relatively uniform and free of significant outliers. Conversely, a substantially larger RMSE indicates the presence of occasional but relatively large deviations that have only a limited influence on the average absolute error. This effect is particularly evident for the DHT22 temperature channel, which exhibits the lowest MAE among all sensing channels but a noticeably larger RMSE due to several isolated transient deviations visible in Figure 16. Consequently, the proposed Wi-Fi communication architecture maintains high transmission accuracy for the vast majority of measurements while preserving overall data integrity across all sensing channels.

Overall, Figure 18 demonstrates that the proposed distributed monitoring system provides reliable wireless data transmission for heterogeneous sensing modules. The remaining discrepancies between the wired and wireless measurements are primarily associated with the intrinsic metrological characteristics and dynamic response of the sensing devices rather than with the wireless communication subsystem itself. Together with the distribution-based analysis presented in Figure 17, these results provide a comprehensive statistical evaluation of the transmission reliability of the proposed monitoring platform.

To complement the point error metrics (MAE and RMSE), a statistical confidence and uncertainty analysis was performed for all sensing channels. The difference between the wireless and the wired measurements was considered for each sensing channel and characterized by two components: the systematic error (bias) and the random error expressed by the standard deviation (SD). The statistical confidence of the estimated bias was evaluated using the 95% confidence interval (CI), whereas the uncertainty of individual wireless measurements relative to the wired reference was assessed using the 95% limits of agreement (LoA) according to the Bland–Altman method. The limits of agreement represent the expected interval within which the majority of differences between the wireless and wired measurements are located and provide an estimate of the expanded uncertainty of the proposed monitoring system.

The resulting statistical confidence measures and uncertainty estimates are summarized in Table 9.

The statistical confidence analysis confirms the conclusions obtained from the error distribution analysis (Figure 17) and the MAE/RMSE comparison (Figure 18). The ultrasonic distance sensors and the DHT22 temperature channel exhibit the smallest systematic bias together with the narrowest confidence intervals and limits of agreement, demonstrating excellent consistency between the wireless and wired measurements. These results indicate that the proposed Wi-Fi communication architecture preserves measurement accuracy while introducing negligible additional uncertainty.

The magnetometer channels exhibit systematic biases close to zero but comparatively wider limits of agreement, indicating that the observed uncertainty is governed primarily by random variability rather than systematic transmission errors. In contrast, the humidity channels present the largest uncertainty intervals, particularly for the DHT22 humidity measurements, reflecting the combined influence of environmental sensitivity and the intrinsic characteristics of low-cost humidity sensors. Nevertheless, the confidence intervals remain well defined, confirming the repeatability and statistical consistency of the acquired measurements.

Overall, the statistical confidence and uncertainty analysis demonstrates that the proposed distributed Wi-Fi monitoring platform provides reliable wireless transmission across heterogeneous sensing channels. The remaining discrepancies between the wired and wireless measurements are primarily attributable to the intrinsic characteristics of the sensing modules rather than to the wireless communication subsystem itself. Together with the MAE, RMSE, and distribution-based analyses, these results provide a comprehensive statistical validation of the proposed structural health monitoring system.

The statistical analyses presented in the previous subsections indicate that the observed differences between the wired and wireless measurements originate primarily from the sensing hardware rather than from the wireless communication architecture. A more detailed examination shows that the overall measurement discrepancy is the result of three principal sources of uncertainty: systematic differences between individual sensor modules, random environmental and measurement variability, and the wireless communication subsystem.

The first source corresponds to systematic inter-sensor differences and is reflected by the measurement bias. Since the wired and wireless measurements were acquired using two independent sensor modules of the same type, each channel is affected by manufacturing tolerances and calibration differences between the individual devices. This effect is particularly evident for the DHT11 sensor, where the average bias reaches approximately −0.84 °C for temperature and −1.62% RH for humidity. In both cases, the Mean Absolute Error is very close to the corresponding bias, indicating that the observed discrepancy is predominantly systematic rather than random. A similar but considerably smaller constant offset is observed for the ultrasonic distance sensors, where the bias remains within approximately −0.043 to −0.047 mm. These observations demonstrate that the principal source of systematic error is the intrinsic variability between individual sensing modules. The most effective approach for reducing this component is individual sensor calibration against a common reference together with offset and gain compensation. The superior performance of the DHT22 temperature channel further confirms this conclusion, as its improved factory calibration reduces the systematic bias to approximately −0.04 °C.

The second source of uncertainty is associated with random variability of the measured physical quantities. This behaviour is most evident for the magnetometer channels, where the systematic bias remains close to zero while the standard deviation is considerably larger. Such behaviour is mainly caused by the high sensitivity of magnetic field measurements to slight differences in sensor position and orientation, as well as to local electromagnetic disturbances. A comparable, although less pronounced, effect is observed for the humidity channels, where local microclimatic variations occasionally produce relatively large measurement deviations. These random fluctuations can be reduced through closer spatial placement of the sensing modules, improved synchronization of the measurement instants, shielding from external electromagnetic interference where appropriate, and the application of short-window digital filtering or averaging algorithms.

The third contribution is associated with the wireless communication subsystem. The experimental results demonstrate that this contribution is negligible under the investigated operating conditions. All sensing modules generate digital measurement data, and all 500 measurement samples were successfully transmitted through the Wi-Fi communication channel without packet loss. Consequently, the wireless transmission process does not introduce measurable numerical distortion into the acquired data, and the remaining discrepancies are primarily governed by sensor characteristics and environmental conditions rather than by the communication architecture itself.

Overall, the error analysis demonstrates that the proposed distributed Wi-Fi monitoring system provides reliable wireless transmission while preserving measurement integrity across heterogeneous sensing channels. Future optimization of the monitoring platform should therefore focus primarily on improving sensor calibration, sensor placement, synchronization strategies, and advanced signal-processing techniques rather than on modifications of the wireless communication subsystem, which exhibited stable and reliable performance throughout the experimental study.

The experimental results presented above primarily evaluate the accuracy and reliability of wireless data transmission by comparing wired and wireless communication channels under controlled measurement conditions. However, practical structural health monitoring systems are expected to operate under continuously changing environmental and operational conditions. Therefore, in addition to the quantitative evaluation of measurement accuracy, a series of supplementary experiments was conducted to assess the robustness of the proposed monitoring platform under representative operating scenarios.

The additional experimental validation focused on two practical aspects frequently encountered during long-term structural monitoring. The first scenario investigated the influence of changing environmental conditions, while the second evaluated the system response to dynamic mechanical loading of the monitored structure. These experiments were intended to verify that the proposed distributed monitoring system maintains reliable operation not only under laboratory conditions but also under representative environmental and operational influences that may occur during real-world deployment.

To further evaluate the robustness of the proposed distributed monitoring system under representative operating conditions, an additional experimental scenario was conducted under naturally changing environmental conditions. Unlike the previous experiments, which primarily assessed the accuracy of wireless data transmission, this validation focused on the ability of the proposed monitoring platform to maintain stable operation during variations in weather conditions.

**Figure 19:**
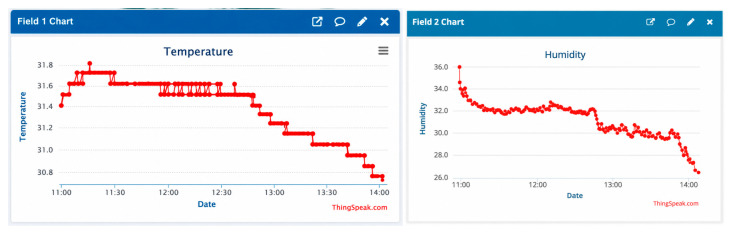
Temperature and relative humidity measurements recorded under changing environmental conditions.

The experiment was performed under continuously changing environmental conditions, including cloudy and rainy weather followed by clear sunny conditions. During the monitoring period, temperature, relative humidity, and ambient illumination were continuously recorded using the integrated environmental sensors. The corresponding temperature and humidity measurements obtained during the experiment are presented in Figure 19.

**Figure 20:**
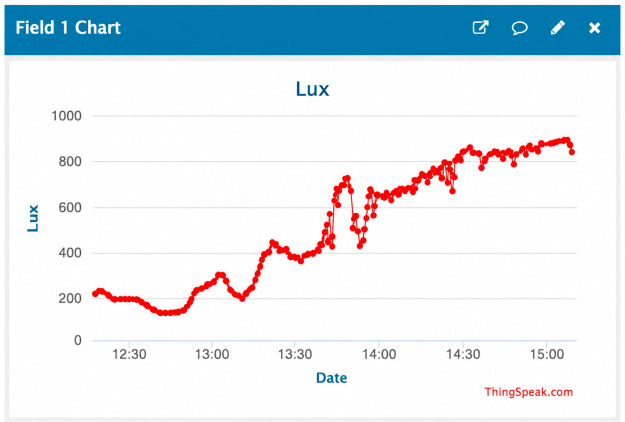
Ambient illumination measurements (TSL2561 sensor) under changing weather conditions.

To verify the correctness of environmental monitoring, ambient illumination was additionally measured using the TSL2561 digital light sensor. The obtained illumination values are presented in Figure 20.

At the beginning of the experiment, corresponding to cloudy and rainy weather, relatively low illumination values were recorded. After precipitation ceased and sunlight intensity increased, the measured illumination gradually increased, reaching a maximum value of approximately 825 lx. The observed illumination profile closely followed the actual weather conditions throughout the monitoring period, confirming the correct operation of both the sensing subsystem and the wireless communication architecture.

Simultaneously, the temperature and humidity sensors continuously recorded changes in the surrounding environment without communication interruptions or missing measurements. The obtained time series remained stable throughout the experiment and consistently reflected the environmental changes occurring during the observation period.

The obtained results demonstrate that the proposed Wi-Fi-based distributed monitoring system maintains stable operation under continuously changing environmental conditions. The successful acquisition and wireless transmission of environmental measurements confirm the suitability of the proposed architecture for long-term deployment in practical structural health monitoring applications, where monitoring systems are expected to operate reliably under varying weather conditions.

To further evaluate the applicability of the proposed monitoring system under representative structural loading conditions, a second experimental scenario was conducted to investigate the response of the distributed monitoring platform to dynamic mechanical loading. The objective of this experiment was to verify the capability of the proposed system to detect changes in the spatial position and dynamic behavior of the monitored structure in real time.

The experiment was performed using a laboratory-scale building model subjected to a series of controlled mechanical excitations. External forces were successively applied to different parts of the structure to induce vibrations and slight changes in its spatial orientation, thereby simulating dynamic loading conditions that may occur during the operation of engineering structures. The response of the structure was continuously recorded using the MPU6050 inertial measurement unit, which integrates a three-axis accelerometer and a three-axis gyroscope.

**Figure 21:**
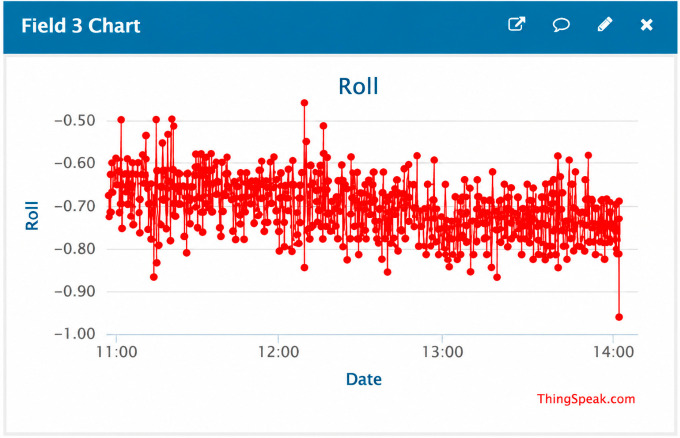
Variation in the measured inclination angle during sequential mechanical loading of the monitored structure.

**Figure 22:**
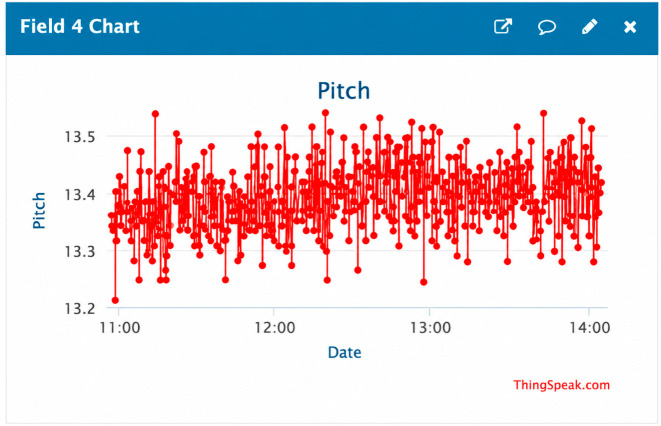
Changes in the spatial orientation of the monitored structure during dynamic excitation.

**Figure 23:**
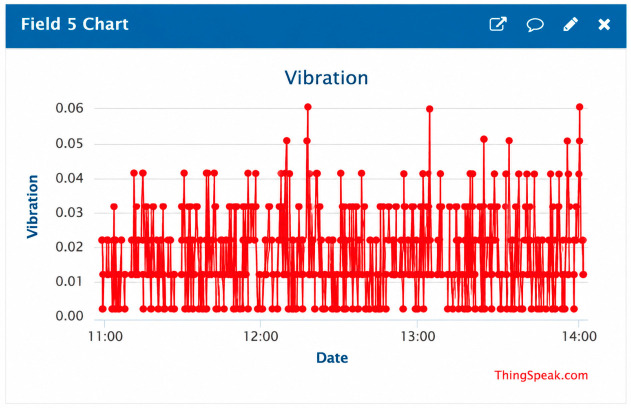
Time history of vibration measurements recorded by the MPU6050 sensor during dynamic loading.

The measured vibration characteristics, inclination angles, and orientation parameters obtained during the experiment are presented in Figure 21, Figure 22 and Figure 23.

During periods without external loading, the recorded signals remained stable, indicating the absence of significant structural movement and confirming the stable operation of both the sensing subsystem and the wireless communication channel.

Following the application of mechanical loads, pronounced variations in vibration amplitude, inclination angle, and spatial orientation were observed. The recorded time series clearly reflected the onset and duration of each mechanical excitation, while the wireless communication system successfully transmitted all measurement data without interruptions or loss of information. The obtained results demonstrate that the proposed monitoring platform is capable of continuously tracking dynamic changes in the structural state and accurately detecting external mechanical disturbances.

The additional experimental validation confirms that the proposed Wi-Fi-based distributed monitoring system maintains reliable operation not only under varying environmental conditions but also under dynamic loading scenarios. These results extend the experimental evaluation beyond communication reliability and demonstrate the practical suitability of the proposed architecture for continuous structural health monitoring of engineering structures subjected to dynamic operational loads.

The experimental results demonstrate that the proposed Wi-Fi-based distributed monitoring system provides reliable transmission of heterogeneous measurement data while preserving the temporal characteristics of the recorded signals. Across all analysed sensing channels, no missing samples or structural distortions of the time series were observed throughout the complete sequence of 500 measurements, confirming the stability of the proposed communication architecture and the integrity of the end-to-end data transmission chain.

Among all sensing channels, the highest agreement between wired and wireless measurements was achieved for the HC-SR04 distance sensors and the DHT22 temperature channel. Their consistently low MAE and RMSE values indicate that wireless communication introduces only negligible transmission errors and does not affect the ability to monitor small structural displacements or temperature variations. These results demonstrate that standard IEEE 802.11 communication is capable of supporting measurement tasks requiring high temporal consistency and reliable preservation of signal dynamics.

Magnetometer measurements exhibit moderately higher deviations than distance measurements while maintaining good agreement between wired and wireless acquisition. The observed variability is primarily associated with the intrinsic characteristics of magnetic field sensing, including sensitivity to electromagnetic interference, sensor orientation, and calibration accuracy, rather than with the wireless communication channel itself. Consequently, the proposed system remains suitable for monitoring spatial trends and dynamic changes in magnetic field behaviour, although applications requiring high-precision absolute measurements may benefit from additional filtering and calibration procedures.

Environmental monitoring channels demonstrate different levels of performance depending on sensor characteristics. The DHT22 provides highly stable temperature measurements and significantly outperforms the DHT11, whereas humidity measurements exhibit the greatest variability throughout the experimental study. Since humidity sensing is inherently influenced by environmental fluctuations, sensor response characteristics, and calibration accuracy, the observed deviations should be interpreted primarily as sensor-related limitations rather than deficiencies of the proposed communication architecture.

The integrated evaluation of all sensing channels confirms that the proposed system successfully preserves diagnostically relevant information despite differences in sensor accuracy and metrological performance. From an engineering perspective, reliable preservation of temporal behaviour and long-term measurement consistency is more important than achieving identical numerical accuracy for all sensing modalities, since practical structural health monitoring systems inherently combine sensors with different physical principles, resolutions, and environmental sensitivities.

Several limitations should nevertheless be considered. First, magnetometer and humidity measurements would benefit from additional calibration and digital filtering procedures to reduce measurement variability. Second, although the ThingSpeak (MathWorks Inc., Natick, MA, USA) cloud platform proved suitable for prototype validation, practical deployment requires dedicated secure server infrastructure together with enhanced cybersecurity mechanisms, user authentication, and protected data management. Finally, comprehensive validation of the proposed monitoring system requires long-term field experiments under realistic environmental conditions and operational loading scenarios.

Overall, the obtained results confirm that the proposed Wi-Fi-based distributed architecture provides a reliable and scalable platform for structural health monitoring. By combining widely available IEEE 802.11 communication technology with distributed sensing and efficient data transmission algorithms, the developed system offers a practical alternative to more complex proprietary monitoring solutions while maintaining satisfactory measurement reliability for long-term engineering applications.

Although the proposed distributed monitoring platform demonstrated reliable communication performance under the investigated conditions, several limitations should be considered.

First, the current experimental validation was performed using a limited number of sensor nodes. Consequently, the influence of significantly increased node density on communication latency, network throughput, synchronization, and overall energy consumption has not yet been comprehensively evaluated. Future studies will therefore focus on large-scale deployment of the proposed architecture on real engineering structures, including bridges, multi-storey buildings, and complex building complexes, in order to investigate communication performance under realistic operating conditions.

Second, although the proposed platform supports heterogeneous sensing devices and distributed wireless communication, its current functionality is primarily focused on reliable data acquisition, transmission, and visualization. Future versions of the system will incorporate additional sensing technologies, optimized energy management strategies, and intelligent communication mechanisms to further improve scalability, reliability, and long-term autonomous operation.

Finally, automated structural condition assessment currently relies mainly on engineering interpretation of the collected measurements. Future research will integrate machine learning and artificial intelligence algorithms for automatic anomaly detection, damage classification, structural degradation prediction, and remaining service life estimation. These enhancements are expected to transform the proposed monitoring platform into a predictive structural health management system capable of supporting intelligent maintenance and decision-making.

## 5. Conclusions

This study presented the development and experimental validation of a distributed structural health monitoring system based on standard IEEE 802.11 Wi-Fi communication. The proposed architecture integrates heterogeneous sensing modules within a unified monitoring framework and combines distributed data acquisition, wireless communication, cloud-based data management, and centralized analysis to support continuous monitoring of engineering structures.

A distributed communication algorithm was developed to coordinate sensor operation, wireless data transmission, and server-side processing. Experimental validation using 500 consecutive measurements demonstrated stable communication performance without data loss or structural distortion of the recorded time series. The highest agreement between wired and wireless acquisition methods was achieved for the HC-SR04 distance sensors and the DHT22 temperature channel, whereas magnetometer and humidity measurements exhibited greater variability owing primarily to sensor characteristics rather than communication-related effects.

The obtained results confirm that standard Wi-Fi technology can provide reliable communication for distributed structural health monitoring while offering the flexibility, scalability, and ease of deployment required for practical engineering applications. The proposed architecture therefore represents a cost-effective alternative to proprietary monitoring systems and can be adapted to other domains requiring distributed environmental or infrastructure monitoring through appropriate modification of the sensing layer.

The principal scientific contribution of this work is the development and experimental validation of a distributed Wi-Fi-based structural health monitoring architecture together with a communication strategy that enables reliable integration of heterogeneous sensing modules within a unified monitoring platform. Future research will focus on long-term field validation, optimization of synchronization and communication strategies, implementation of enhanced cybersecurity mechanisms, and evaluation of network performance under large-scale deployment conditions.

The proposed distributed Wi-Fi-based monitoring platform establishes a foundation for the further development of scalable structural health monitoring systems. Future research will focus on extending both the communication capabilities and the practical applicability of the proposed architecture.

One of the primary directions will be large-scale field deployment involving a substantially greater number of distributed sensor nodes installed on real engineering structures, including bridges, multi-storey buildings, and industrial facilities. Such experiments will enable comprehensive evaluation of communication reliability, network scalability, synchronization stability, and energy consumption under long-term operating conditions and varying environmental influences.

Another important research direction is the integration of additional sensing technologies. Future versions of the system will incorporate strain gauges, vibration sensors, tilt sensors, fiber Bragg grating (FBG) sensors, GNSS receivers, and other specialized structural monitoring devices, thereby extending the range of measurable structural parameters and improving diagnostic capabilities.

Further development will also address the integration of the proposed platform with Building Information Modeling (BIM), Digital Twin technologies, and cloud-based monitoring services to support real-time visualization, centralized data management, and decision support throughout the entire lifecycle of engineering structures.

Finally, future research will focus on the application of artificial intelligence and machine learning techniques for automated anomaly detection, damage classification, prediction of structural degradation, and remaining service life estimation. The combination of distributed wireless sensing and intelligent data analysis is expected to transform the proposed system from a monitoring platform into a predictive structural health management framework capable of supporting maintenance planning and risk-informed decision making.

From a practical perspective, the proposed distributed monitoring platform can be applied to long-term monitoring of bridges, buildings, industrial facilities, and other civil engineering structures where reliable wireless communication and scalable sensor deployment are required. Nevertheless, large-scale validation under real operating conditions and further optimization of energy efficiency and intelligent data processing remain important challenges that will be addressed in future research.

## Figures and Tables

**Table 1:** Comparison of representative distributed structural health monitoring systems with the proposed monitoring architecture.

Feature	Proposed System	Sun et al. [20]	Dolińska et al. [21]	Su et al. [22]	Yang et al. [23]	Szagri et al. [24]	Duobiene et al. [25]
Primary application	Structural health monitoring	Vibration monitoring	Historic building monitoring	Smart home monitoring	Review of SHM systems	Environmental monitoring	Environmental monitoring
Distributed architecture	Yes	Yes	Yes	Partial	Review	Yes	Partial
Communication architecture	Wi-Fi Mesh/ad hoc	WSN	LoRaWAN	Wi-Fi Multihop	Various	Wi-Fi	Wi-Fi
Heterogeneous sensor integration	Yes	No	Partial	Partial	Review	Partial	Partial
Structural parameter monitoring (cracks/displacement)	Yes	No	Yes	No	Yes	No	No
Mathematical network model	Yes	No	No	No	No	No	No
Distributed communication algorithm	Yes	No	No	No	No	No	No
Statistical validation (MAE/RMSE)	Yes	No	No	No	No	No	No
Experimental validation	Yes	Yes	Yes	Yes	Review	Yes	Yes
Scalability	High	Moderate	High	Moderate	High	Moderate	High
Fault tolerance	High	Partial	Partial	High	Partial	High	Partial

**Table 2:** Quantitative comparison of wireless communication technologies for structural health monitoring applications.

Parameter	BLE 5.x	ZigBee	LoRaWAN	Wi-Fi (IEEE 802.11n/ac)
Operating frequency	2.4 GHz	2.4 GHz (868/915 MHz optional)	Sub-GHz (433/868/915 MHz)	2.4/5 GHz
Typical data rate	1–2 Mbps	250 kbps	0.3–50 kbps	72–600+ Mbps
Typical communication range	10–100 m	10–100 m	2–15 km	30–100 m
Network topology	Star, Mesh	Mesh	Star-of-stars	Infrastructure, Mesh, Ad hoc
Typical latency	10–100 ms	15–100 ms	>500 ms	1–10 ms
Power consumption	Very low	Very low	Ultra-low	Moderate–High
Support for high-frequency sampling	Moderate	Moderate	Poor	Excellent
Transmission of heterogeneous sensor data	Moderate	Good	Limited	Excellent
Real-time monitoring capability	Moderate	Good	Poor	Excellent
Compatibility with IP networks	Moderate	Limited	Good (via gateway)	Native
Suitability for distributed SHM	Moderate	Good	Limited	Excellent

**Table 3:** Experimental setup and operating parameters of the proposed distributed monitoring system.

Parameter	Value
Number of sensor nodes	4
Wireless protocol	IEEE 802.11 Wi-Fi
Wi-Fi module	ESP32
Router	MikroTik hAP ax3
Radio bridge	MikroTik RBLHGG-60ad
Communication mode	Client mode
Cloud platform	ThingSpeak
Number of measurements	500
Transmission protocol	HTTPS
Sampling strategy	Periodic
Experimental duration	~3 h
Power-saving mode	Deep Sleep

**Table 4:** Hardware components of the proposed distributed structural health monitoring system.

Component	Monitored Parameter	Main Characteristics	Interface	Rationale for Selection
HC-SR04	Crack propagation/structural displacement	Measuring range: 2–400 cm; resolution ≈3 mm	GPIO	Non-contact distance measurement, low cost, straightforward integration with ESP32
MPU-6050 (GY-521)	Acceleration and angular velocity	6-DoF MEMS inertial measurement unit	I^2^C	Reliable inertial measurements with low computational complexity
MPU-9250	Acceleration, angular velocity, magnetic field	9-DoF MEMS IMU with integrated AK8963 magnetometer	I^2^C/SPI	Simultaneous inertial and magnetic field monitoring, gyroscope drift compensation
DHT22	Temperature and humidity	Factory calibrated; operating range −40 to 80 °C; 0–100% RH	Single-wire digital	Stable environmental monitoring with improved measurement accuracy
ESP32	Data processing and wireless communication	Dual-core 240 MHz MCU; IEEE 802.11 b/g/n Wi-Fi	Wi-Fi, GPIO, I^2^C, SPI	Integrated wireless communication, sufficient processing capability, low power consumption

**Table 5:** Comparative analysis with existing SHM architectures.

Architecture	Communication Type	Data Processing	Scalability	Reliability
Centralized wired SHM [42]	Wired	Centralized	Low	Medium
ZigBee-based WSN SHM [43]	IEEE 802.15.4	Partially distributed	Medium	Medium
Cloud IoT SHM [44]	Wi-Fi/LPWAN	Centralized	High	Medium
Proposed architecture	Wi-Fi Mesh	Edge + Distributed	High	High

**Table 6:** Comparison of SHM network architecture with classical MANET architectures.

Parameter	AODV [45]	OLSR [46]	DSR [47]	Proposed Architecture
Type	Reactive	Proactive	Reactive	Hybrid
Energy awareness	No	No	No	Yes
Scalability	Medium	High	Medium	High
QoS support	Limited	Limited	None	Yes
Convergence	Medium	Fast	Slow	Fast
Suitability for SHM	Partial	Partial	No	Yes

**Table 7:** Network devices used in the proposed distributed structural health monitoring system and their functions.

Device	Function in the Proposed System	Key Advantage
ESP32	Sensor node controller and wireless communication	Integrated Wi-Fi connectivity and local data processing
MikroTik RBLHGG-60ad	Backbone point-to-point wireless communication	High-throughput, low-latency transmission over long distances
MikroTik hAP ax3	Edge gateway and traffic aggregation	Reliable routing and communication management
ThingSpeak Cloud	Data storage, visualization, and remote access	Cloud-based monitoring and rapid experimental deployment

**Table 8:** Operating modes of the NodeMCU ESP8266 wireless communication module.

Operating Mode	Wi-Fi Module	CPU Status	RTC	Typical Current Consumption	Application in SHM
Active Mode	Enabled	Active	Enabled	Highest power consumption	Continuous data acquisition and transmission
Modem Sleep	Disabled	Active	Enabled	15.2–16.2 mA	Data processing without wireless communication
Light Sleep	Disabled	Sleep	Enabled	0.55–1.8 mA	Short idle periods between transmissions
Deep Sleep	Disabled	Disabled	Enabled	20 μA	Long-term autonomous monitoring with periodic data transmission

**Table 9:** Statistical confidence and uncertainty analysis of the wireless-versus-wired measurements.

Measurement Channel	Bias	SD	95% CI for Bias	95% Limits of Agreement (LoA)
Distance 1 (mm)	−0.043	0.015	[−0.044, −0.042]	[−0.071, −0.014]
Distance 2 (mm)	−0.047	0.011	[−0.048, −0.046]	[−0.069, −0.026]
Magnetometer X (µT)	−0.158	0.754	[−0.224, −0.092]	[−1.635, 1.320]
Magnetometer Y (µT)	0.048	0.637	[−0.008, 0.104]	[−1.201, 1.298]
Magnetometer Z (µT)	0.092	0.675	[0.033, 0.151]	[−1.230, 1.415]
DHT11 Temperature (°C)	−0.842	0.206	[−0.860, −0.824]	[−1.245, −0.439]
DHT22 Temperature (°C)	−0.041	0.096	[−0.049, −0.032]	[−0.229, 0.148]
DHT11 Humidity (%RH)	−1.620	0.695	[−1.681, −1.559]	[−2.982, −0.259]
DHT22 Humidity (%RH)	−1.169	1.751	[−1.323, −1.016]	[−4.601, 2.262]

## Data Availability

The data presented in this study are available on request from the corresponding author.

## Abbreviations

**Abbreviation**	**Definition**
ADC	Analog-to-Digital Converter
AODV	Ad hoc On-Demand Distance Vector
BLE	Bluetooth Low Energy
CSI	Channel State Information
CSMA/CA	Carrier Sense Multiple Access with Collision Avoidance
DCU	Data Collection Unit
DHT	Digital Humidity and Temperature Sensor
DMP	Digital Motion Processor
DSR	Dynamic Source Routing
EMU	External Monitoring Interface
GSM	Global System for Mobile Communications
HTTP	Hypertext Transfer Protocol
HTTPS	Hypertext Transfer Protocol Secure
I^2^C	Inter-Integrated Circuit
IDE	Integrated Development Environment
IMU	Inertial Measurement Unit
IoT	Internet of Things
IP	Internet Protocol
LED	Light-Emitting Diode
LPWAN	Low-Power Wide-Area Network
LTE	Long-Term Evolution
MAE	Mean Absolute Error
MANET	Mobile Ad hoc Network
MCU	Microcontroller Unit
MEMS	Micro-Electro-Mechanical Systems
MO	Monitored Object
MPU	Motion Processing Unit
OFDMA	Orthogonal Frequency-Division Multiple Access
OLSR	Optimized Link State Routing
OSI	Open Systems Interconnection
OTP	One-Time Programmable
RAM	Random Access Memory
RMSE	Root Mean Square Error
RSSI	Received Signal Strength Indicator
SDK	Software Development Kit
SHM	Structural Health Monitoring
SNR	Signal-to-Noise Ratio
SPI	Serial Peripheral Interface
SU	Sensor Unit
TCP	Transmission Control Protocol
T/R Server	Transceiver Server
UMTS	Universal Mobile Telecommunications System
Wi-Fi	Wireless Fidelity (IEEE 802.11 Wireless Networking)
WSN	Wireless Sensor Network
